# Relative motion splints versus metacarpophalangeal joint blocking splints in the management of trigger finger: Study protocol for a randomized comparative trial

**DOI:** 10.1371/journal.pone.0307033

**Published:** 2024-08-13

**Authors:** Li Xian Leong, Siaw Chui Chai, Julianne W. Howell, Hanif Farhan Mohd Rasdi, Nur Rahimawati Abdul Rahman

**Affiliations:** 1 Faculty of Health Sciences, Occupational Therapy Programme, Centre for Rehabilitation & Special Needs Studies, Universiti Kebangsaan Malaysia, Kuala Lumpur, Malaysia; 2 Occupational Therapy Unit, Hospital Sultan Haji Ahmad Shah, Ministry of Health of Malaysia, Putrajaya, Malaysia; 3 Self-Employed Hand and Upper Extremity Therapy Consultant, Saint Joseph, Michigan, United States of America; 4 Orthopaedic Department, Hospital Sultan Haji Ahmad Shah, Ministry of Health of Malaysia, Putrajaya, Malaysia; Brunel University London, UNITED KINGDOM OF GREAT BRITAIN AND NORTHERN IRELAND

## Abstract

**Background:**

Evidence supports the use of hand-based metacarpophalangeal joint (MCPJ) blocking splints as an intervention for trigger finger (TF). In practice, finger-based relative motion (RM) splints are also implemented without evidence.

**Purpose:**

This randomized comparative trial (RCT) aims to evaluate implementation of MCPJ blocking and RM splints for effectiveness, function, occupational performance and wearability after 6 weeks of TF management.

**Methods and analysis:**

Priori analysis determined 36 individuals were needed for random assignment to the RM or MCPJ blocking splint groups. Individuals must be aged ≥21 years, and diagnosed with TF involving ≥1 finger. For blinding purposes, the primary author screens for eligibility, fabricates the splints and educates. Therapist A administers the primary outcome measures Week-1 and Week-6—stage of stenosing tenosynovitis and secondary outcome measures- number of triggering events in 10 active fists, visual analog scales (VAS) for pain, splint comfort and satisfaction, Disabilities of the Arm, Shoulder and Hand, and Canadian Occupational Performance Measure. Therapist B in Week-3 instructs participants in deep tissue massage and administers splint wearability VASs. The RM pencil test is used to determine the affected finger(s) MCPJ splint position i.e., more extension or flexion based on participant response. The MCPJ blocking splint holds the MCPJ in a neutral position. Analysis involves a mixed-effects ANOVA to compare Week-1 and Week-6 primary and secondary outcomes.

**Results:**

Recruitment and data collection are ongoing.

**Discussion:**

Biomechanically RM splints control tendon excursion and reduce passive tendon tension while allowing unencumbered finger motion and hand function. Hence clinicians use RM splints as an intervention for TF, despite the lack of implementation evidence. This RCT implements a function-focused as well as patient-centered approach with partial blinding of assessors and participants.

**Conclusion:**

We anticipate that this study will provide evidence for the implementation of RM splints to manage adults with TF.

**Trial registration:**

**Clinical trial registration** This trial is registered with ClinicalTrials.gov (NCT05763017).

## Introduction

Trigger finger (TF) is a common condition that interferes with gliding of the digital flexor tendon and tendon sheath through the pulley system of the finger [1]. This condition may result in pain, clicking, catching and loss of motion, and is thought to be due to inflammation with the most common site near the metacarpophalangeal joint (MCPJ) at the first annular pulley (A1) [2]. TF can affect activities of daily living, notably hand functions that require precision, speed, gross power grip, 3-jaw chuck pinch, and manipulation [3,4] and for some, TF has been reported to contribute significantly to disability [3].

Interventions for TF can include surgery [2], corticosteroid injection [1] or splinting [4]. Generally splinting and/or injection are the first line of treatment [5]. The primary purpose for using a splint is to limit flexor tendon and sheath excursion through the A1 pulley [6]. Various hand-based and finger-based splints have been described [7]. These splints usually immobilize one finger joint, either the MCPJ, proximal interphalangeal joint (PIPJ), or distal interphalangeal joint (DIPJ). Per review of the literature [7], the most frequently cited type of splint blocks the MCPJ [6,8–13], while others block the PIPJ [8,14,15] or the DIPJ [10,16]. When compared with a DIPJ blocking splint, Tarbhai et al. [10] noted that the MCPJ blocking splint was preferred by patients because it felt more stable, comfortable, and their fingers were less stiff. These authors [10] reported the DIPJ blocking splint to be troublesome because it slipped off the finger easily and interfered with fingertip prehension.

Another finger-based splint which holds potential for managing TF is the relative motion (RM) splint. Originally designed to protect extensor tendon repairs of the fingers [17], the RM splint is low profile [17], small in size [18], easy to fabricate [18], and allows full mobility of the fingers minus 20–25° of MCPJ motion [19]. RM splints are named by the direction in which the affected finger’s MCPJ is positioned, either in greater extension (RME) or more flexion (RMF) [19]. The extensor tendon repair literature suggests RM splints support hand function [17,18,20], which can contribute to patient adherence [18]. Clinician experts advocate the use RM splints to manage TF [19] although evidence is lacking.

For this study, we are comparing a MCPJ blocking splint and a RM splint that limits motion of the MCPJ of the involved finger(s) by at least 20–25°.

We propose two reasons why RM splints may work as an intervention for TF at the A1 pulley: 1) the position of the affected finger in a RME splint decreases passive tension of the extensor tendon(s), which biomechanically lessens the force of flexion during active finger motion [21,22] and subsequently reducing the force of flexion exerted on the pulley system; and 2) the design of the RME and RMF splints limits MCPJ motion by at least 20–25° to reduce excursion of the flexor tendon and sheath through the A1 pulley without the need to fully block MCPJ motion [23,24].

### Objectives

The primary objective of this study is to compare the effectiveness of wearing either a RM or a MCPJ blocking splint for 6 weeks on therapist-observed signs and patient-report of symptoms. The secondary aim is to compare the effect of these two splints on the patient- report of hand function, occupational performance, splint comfort and satisfaction after 6-weeks of wear.

## Method

### Research design

**Fig 1:**
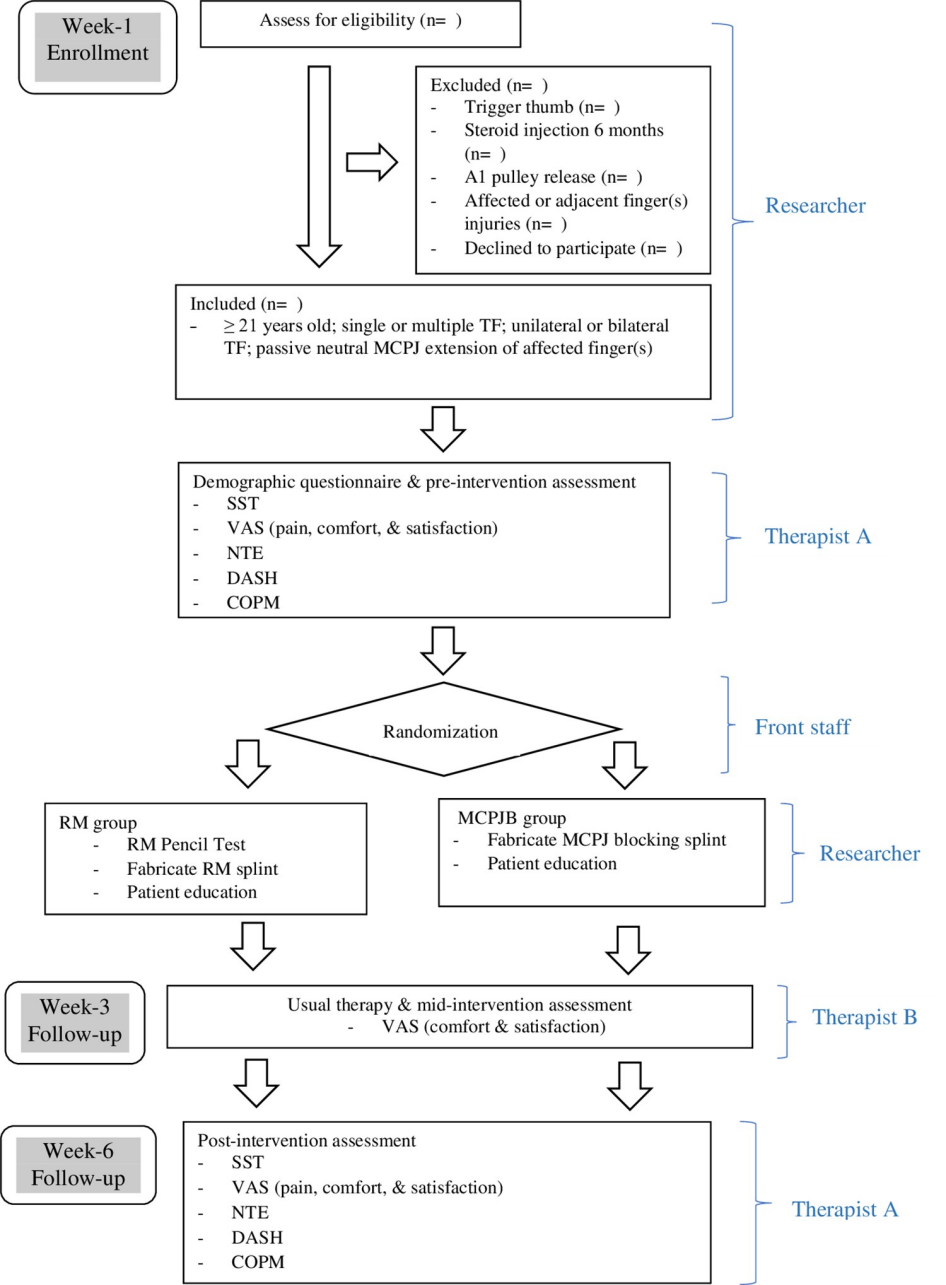
Flow of study per SPIRIT guidelines. Abbreviations: TF—Trigger finger; MCPJ—metacarpophalangeal joint; SST—Stages of Stenosing Tenosynovitis; VAS—Visual Analogue Scale; NTE—number of triggering events in ten active fists; DASH—Disabilities of the Arm, Shoulder and Hand outcome measure; COPM—Canadian Occupational Performance Measure and RM—relative motion.

**Fig 2:**
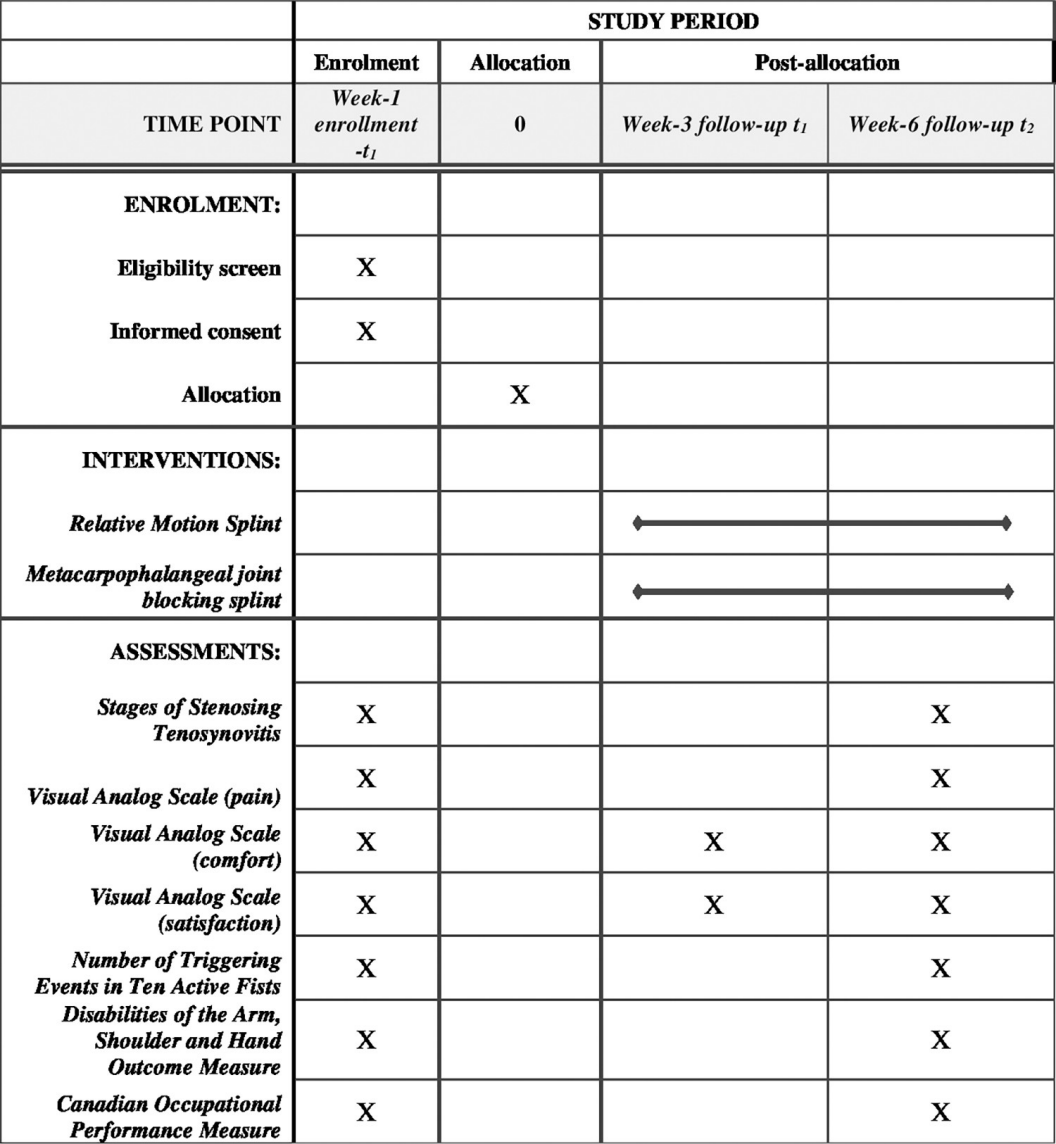
Standard Protocol Items: Recommendations for Interventional Trials (SPIRIT).

This randomized comparative trial (RCT) allocates participants into 2 splint groups: RM and MCPJ blocking. The RCT protocol is guided by the Standard Protocol Items: Recommendations for Interventional Trials (SPIRIT) [25] checklist consisting of a 33-items (S1 File). The flow of study begins with participant enrollment, splint group allocation, Week-1 pre-intervention assessment, splinting intervention, Week-3 mid-intervention assessment and deep tissue massage intervention and Week-6 post-intervention assessment as detailed in Figs 1 and 2.

### Participants: Recruitment and eligibility criteria

Potential participants are recruited from Hospital Sultan Haji Ahmad Shah, Temerloh, Pahang, Malaysia. Those individuals diagnosed by an orthopedic surgeon with TF involving the A1 pulley of the finger(s) and referred to the Hospital’s Occupational Therapy Unit. For those referred to be included, the following criteria must be satisfied: at least 21 years old; one or more trigger fingers; one or both hands involved; and passive MCPJ extension to neutral of the involved finger(s). Potential participants are excluded for the following reasons:

- trigger finger involving the thumb
- steroid injection involving the affected finger within the previous 6 months
- prior surgical release of the A1 pulley involving the affected finger
- a history of fracture, tendon or nerve injury, Dupuytren’s, other soft tissue injuries involving the affected or adjacent fingers

### Sampling size

G*Power was used to compute statistical power analyses and to determine the sample size. Based on a prior TF study [14] which reported a pre-intervention pain score of 4.65 (SD 2.39) and post-intervention pain score of 3.40 (SD 2.44), we calculated the Cohen-d effect size to be 0.518. The Cohen-d effect size was converted to a Cohen-f to determine the sample size needed for this mixed-effect ANOVA study. After digital conversion, the Cohen-f effect size was 0.259. With a Cohen-f effect size of 0.26, confidence level of 95%, significant level of 5%, and statistical power of 80%, 32 participants are required. Since a 10% dropout rate is anticipated, a total of 36 participants are needed with 18 patients allocated to the RM group and 18 patients to the MCPJ blocking group.

### Randomization

A simple random method is used to assign participants to a splint group on Week-1 by a protocol-trained front staff person. This person selects one envelope from a group of sealed envelopes each containing a sheet of paper that designates the participant’s assignment to the RM or the MCPJ blocking group (Fig 1).

### Blinding

Eligibility screening of potential participants is done by the Researcher (primary author LXL) prior to allocating the person to any splint group (Fig 1). This strategy preserves the blinding of Therapist A and the participant during Week-1 pre-intervention assessment. Once the participant is assigned to a splint group, Therapist A, the participant, and the Researcher are no longer blinded to the type of splint. Given the nature of the study and the visibility of splint wearing, ensuring blinding of participants and assessors in splinting studies is nearly methodologically impossible [26]. Given the participant information sheet does not specify which splint group serves as the experimental treatment or comparator group, participants are considered partially blinded.

### Research team

The research team consists of three occupational therapists (the first, second, and fourth authors), a certified hand therapist (the third author), and an orthopedic surgeon (the fifth author). Besides, the study also includes a front office staff person and two Occupational Therapists-Therapist A and Therapist B during the data collection process (Fig 1). Eligibility screening and splint fabrication is done by the Researcher (the first author). Therapist A administers Week-1 pre-intervention and Week-6 post-intervention assessments (Fig 1). Therapist B provides deep tissue massage intervention for each participant and performs the Week-3 mid-intervention assessment (Fig 1).

### Informed consent, registration, baseline assessment and group allocation

Once the person’s eligibility is confirmed, the Researcher issues the participant information sheet (S1 File) and explains the flow of the study (Fig 1). If the person agrees to participate, he/she is assigned an anonymous identity number and asked to sign the informed consent form. Therapist A then administers the demographic questionnaire (S2 File) and completes the Week-1 pre-intervention assessment. Lastly, on Week-1, the participant is randomly assigned by a front office staff person to the RM or MCPJ blocking group (Fig 1).

### Splint fabrication, comfort and satisfaction rating

**Fig 3:**
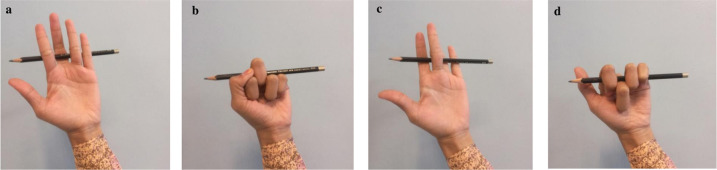
Relative motion pencil test. a) RM extension (RME) fingers open, b) RME fingers closed, c) RM flexion (RMF) fingers open and d) RMF fingers closed.

Participants assigned to the RM group have either a RME or RMF splint fabricated by the Researcher (Fig 1). The design of the RM splint depends on the results of the pencil test [27] conducted by the Researcher (Fig 3). The pencil test is used in conjunction with active finger flexion and extension to assess which position (more extension or flexion) of the MCPJ of the affected finger(s) that best reduces the participant’s reported of symptoms [27]. For the RME pencil test, the Researcher weaves a pencil under the proximal phalanx of the affected finger(s) and over the dorsum of the proximal phalanx of the adjacent fingers [28]. For the RMF pencil test, the Researcher weaves the pencil over the dorsum of the proximal phalanx of the affected finger(s) and under the proximal phalanx of the adjacent fingers [28]. With the pencil in place, for this study the Researcher asks the participant to 1) actively open and close his/her fingers several times and 2) compare the effect of the RME and RMF pencil test on his/her symptom(s). If the participant’s answer is the RME pencil test position, then a RME splint is fabricated. Conversely, if the participant’s answer is the RMF pencil test position, then a RMF splint is made. In using the RM pencil test for assessment, the trial and error splint fabrication method to determine which RM splint design (RME or RMF) is eliminated.

**Fig 4:**
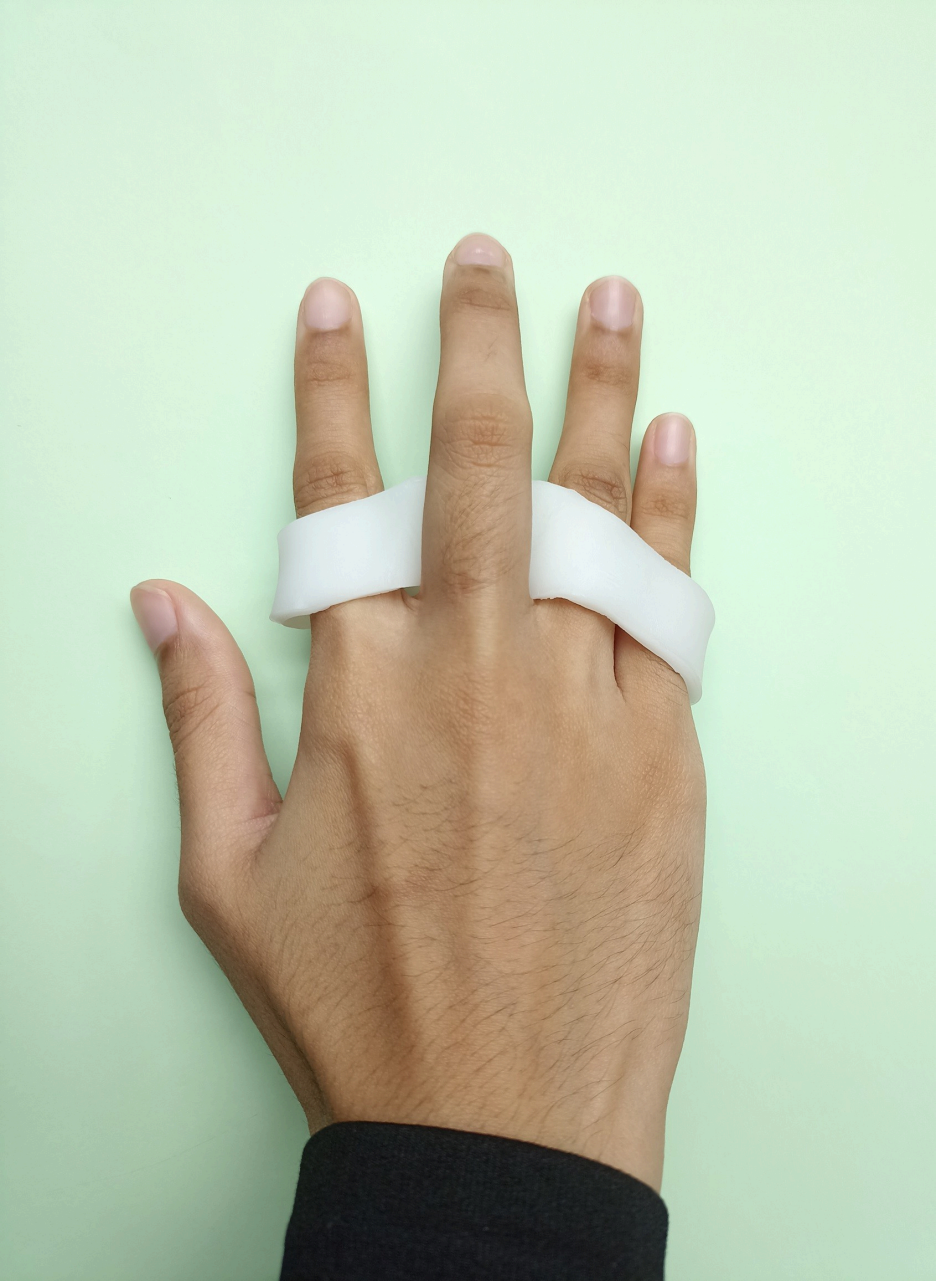
Relative motion extension (RME) splint.

**Fig 5:**
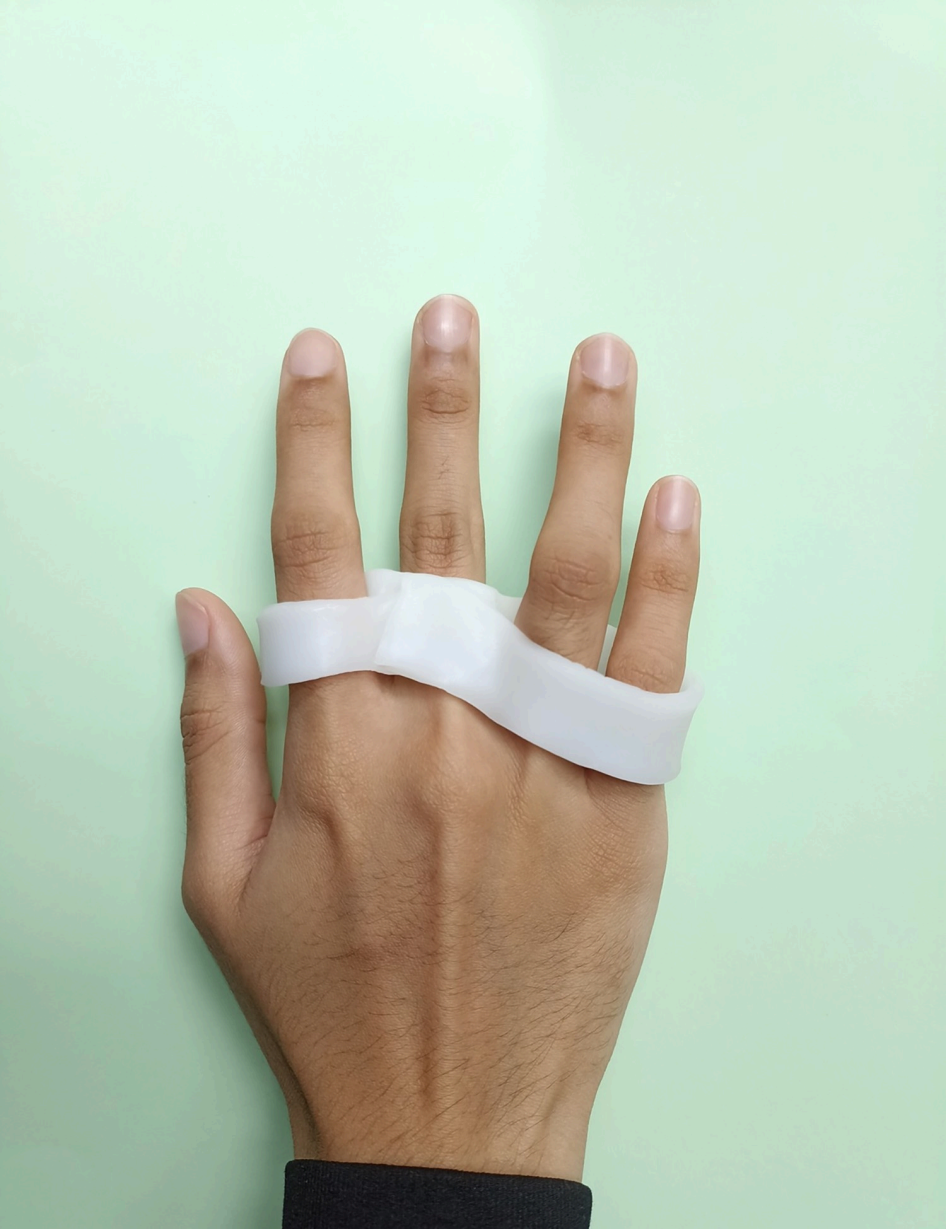
Relative motion flexion (RMF) splint.

Each RME splint includes 4 fingers and is fabricated from a 3.2mm sheet of thermoplastic. The cut width is approximately three quarters the length of the proximal phalanx and the cut length is the circumference measured around the base of all 4 proximal phalanges. For the 4-finger RME splint, the cut strip of thermoplastic is woven around the proximal phalanx of all fingers with the MCPJ of the affected finger(s) positioned in approximately 20–25° more extension than MCPJ of the adjacent fingers (Fig 4). For the 4-finger RMF splint, the thermoplastic is cut the same way but the thermoplastic strip is woven in reverse with the MCPJ of the affected finger(s) positioned in approximately 20–25° more flexion (Fig 5). As necessary, the differential angle of the MCPJ in either flexion or extension may be increased, if 20–25° flexion/extension is not sufficient to reduce/eliminate the participant-reported symptom(s).

**Fig 6:**
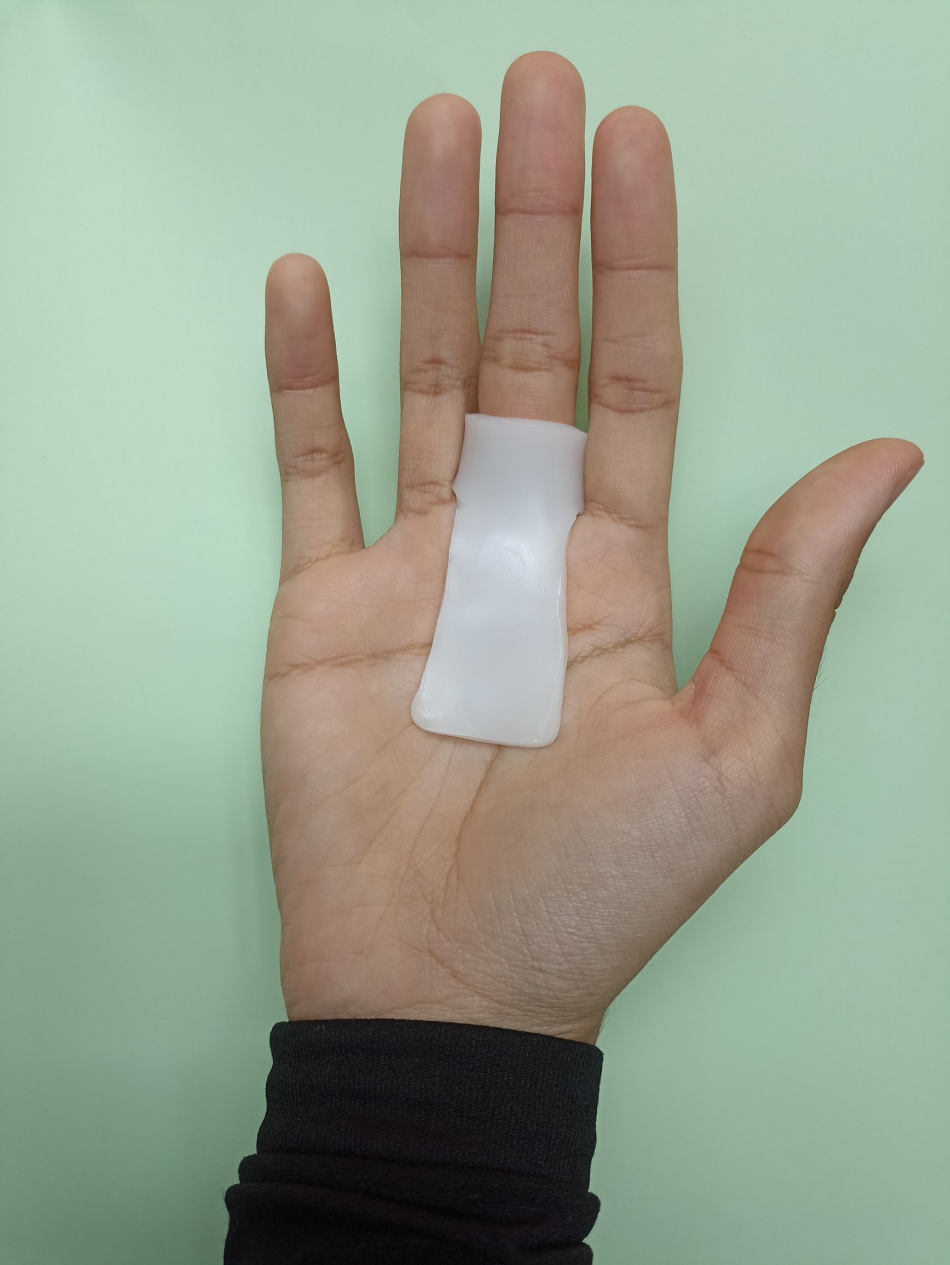
Metacarpophalangeal joint (MCPJ) blocking splint.

To fabricate the MCPJ blocking splint, a T-shape pattern is cut from the sheet of 3.2 mm thermoplastic. The vertical line of T is molded to contour the palm over the volar aspect of the MCPJ, while the horizontal line of T is formed into a circumferential “ring” around the proximal phalanx of the affected finger(s) taking care not to block PIPJ motion (Fig 6).

After either the RM or MCPJ blocking splint is fabricated, the participant is asked to wear it for 15 minutes to ensure the fit is good with no pressure points. If there is pain and/or skin irritation, the splint is modified and the process repeated. Once the participant and therapist are satisfied with the fit of the splint, the participant completes Week-1 VAS for splint comfort and splint satisfaction (S2 File) (Fig 1).

### Instruction and education of participants

All participants will have the usual therapy instruction and education provided. Each participant is instructed by the Researcher to wear their splint full time, 24 hours per day, every day and night for 6 weeks (Fig 1). During this time, the participant is advised to avoid finger motion that exacerbate their symptoms. Each participant was asked to keep a daily diary to report the number of hours the splint was worn during a 24-hour day and to list task(s) for which the splint is removed (Fig 1).

Each participant is educated by the Researcher about the condition of TF including symptoms, cause, risk factors, and possible future interventions if splinting is not successful. Each participant is encouraged to modify or avoid activities that provoke his/her TF symptoms while in or out of the splint. Each participant is asked to contact the Researcher regarding splint fit issues or if the splint has been lost so that the problem can be addressed and documented.

### Therapy intervention and follow up

Each eligible participant is required to attend 3 sessions of therapy, Week-1, Week-3, and Week-6 for this 6-week study (Fig 1). During Week-3, Therapist B administers the splint satisfaction and comfort assessments and instructs participants in deep circular massage over the A1 pulley with the splint removed. After this instruction, the participant is directed to do the deep tissue massage for 5 minutes, 3 times per day at home. If at this time the splint requires modification, Therapist B will assist with this. On Week-6, Therapist A administers the post-intervention assessments to each participant (Fig 1).

### Outcome measures

This RCT is using the severity of triggering as the primary outcome measure to compare the effectiveness of the RM and MCPJ blocking splints. Severity is graded with the stages of stenosing tenosynovitis (SST) classification system [11]. The grading system is divided into six stages: Stage 1: normal finger movement; Stage 2: uneven finger movement, Stage 3: triggering, clicking or catching, Stage 4: finger locked in flexion or extension that can be unlocked by active finger movement, Stage 5: finger locked in flexion or extension that requires passive force to unlock, and Stage 6: finger locked in flexion or extension [11]. Therapist A will assess and record the stage of severity of triggering on Week-1 and Week-6.

Patient-report of pain is a secondary outcome measured using a visual analog scale (VAS). For this study, the 10cm VAS is labelled ‘no pain’ on the left end of the scale and ‘extreme pain’ on the other end. Week-1, Therapist A asks each participant to rate their pain by marking the VAS line and measures the distance in centimeters from the mark to the left end of the line. The participant’s perception of splint wearability i.e., comfort and satisfaction are assessed using two separate VASs. For comfort, the left end is labelled ‘not at all comfortable’ and the other end ‘extremely comfortable’. For satisfaction, the left end is labelled ‘not at all satisfied’ and the other end ‘extremely satisfied’. Participants are asked to mark each scale regarding their splint wear comfort or satisfaction. The centimeter distance from the left end of the scales to the marks are recorded as the splint comfort and satisfaction scores. The wearability VASs are administered and documented by Therapist A on Week-1 and Week-6, by Therapist B on Week-3 (Fig 1).

Another secondary outcome measure to assess frequency of triggering is the number of triggering events in ten active fists (NTE) [29]. The number of triggering events is determined by Therapist A who counts and records the number of times the participant’s finger triggers during 10 repetitions of full active opening and closing of the fingers [29] (Fig 1). If at any time the finger locks during this test, Therapist A will automatically assign a score of 10/10. If multiple fingers are involved, only 10 finger opening and closing will be required and the score will be that of the worst finger.

The Disabilities of the Arm, Shoulder and Hand (DASH) outcome measure is used to assess hand function. The DASH is a 30-item, self-report questionnaire designed to assess the participant’s health status during the previous week and consists of items that assess performance of select activities (21 items), severity of symptoms (5 items) and the impact of the problem on social function, work, sleep and self-image (4 items). There are two optional modules specific to work, sports and/or performing arts that are not being used in this study. The DASH has a total possible score of 100 with a higher score indicating more disability or poorer hand function [30]. Therapist A administers and records the DASH score on Week-1 and Week-6.

The Canadian Occupational Performance Measure (COPM) is used as a secondary outcome measure to compare the effect of each splint on the participant’s occupational performance [31]. Therapist A interviews and records participant’s answers on Week-1 and Week-6 to identify his/her occupation performance problem(s). Each participant is asked to prioritize activities and use a 10-point scale to rate performance and satisfaction. A higher number indicates a better performance and satisfaction rating.

### Statistical analysis

Descriptive statistics will be used to summarize the demographic data including age, gender, occupation, hand dominance, digit(s) involved, associated medical conditions, and duration of triggering. Data will be analyzed and described in total numbers, mean, and the pattern of data. Descriptive statistics will also be used for the primary outcome measures of SST; and secondary outcome measures including DASH, COPM, VAS (pain, comfort, and satisfaction) and NTE. A mixed-effects ANOVA will be used to compare the splint groups Week-1 and Week-6 SST, NTE, VAS (pain, comfort and satisfaction), DASH and COPM data. The minimal dataset is shown in the S3 File.

### Monitoring, ethics and dissemination of information

Potential participants for this study are recruited from a public hospital and monitored by the Clinical Research Centre (CRC) Pahang, Malaysia, a research institute under the guise of the National Institutes of Health, Ministry of Health Malaysia. Officers of the CRC conduct visits to monitor the study and issue reports according to the Centre’s protocol.

This study has been registered on ClinicalTrials.gov with the identifier NCT05763017. Ethics approval was obtained from the Medical Research and Ethics Committee of Ministry of Health (NMRR ID-22-00204-BWM) on 11^th^ May 2022 and the Research Ethics Committee of Universiti Kebangsaan Malaysia (JEP-2022-319) on 15^th^ July 2022.

Prior to becoming a study participant, each patient is given and asked to read an information sheet (S2 File) describing the purpose and design of the study. To participate, an informed consent is signed.

Each participant’s name is kept in a password-protected database, linked only by a study identification number and used on all participant data sheets. The data from this study will be made into a report and disseminated to Hospital Haji Ahmad Shah, the Ministry of Health, and Universiti Kebangsaan Malaysia. Once the data are gathered and the research completed, the raw materials will be stored in the locked file cabinet in the office of the Researcher. Electronic data will be secured and only accessible by a password known to the Researcher. After 5 years from study completion, all materials will be shredded and discarded by the Researcher.

The trial findings will be reported according to the Consolidated Standard of Reporting Trials (CONSORT) guidelines. The manuscript of this trial will be submitted to a peer-reviewed international scientific journal for publishing. The findings may be presented at national conferences and scientific meetings.

## Results

This study was initiated on 28^th^ June 2022 and is currently in the data collection phase where recruitment of participants and implementation of the intervention is ongoing.

## Discussion

Therapists who manage patients with hand and upper limb conditions aim to optimize the patient’s function and ability to participate in all areas of their life. Therapy intervention in this study involves two different splints designed to lessen/eliminate the symptoms of TF with minimal impact on hand function and lifestyle. Splinting with or without steroid injection is described as the first line of intervention for TF with surgery a later option if needed [5]. This study is important as the results will help to fill the knowledge gap identified by literature review [4,7] regarding the role of splinting as an intervention for TF. Three areas were identified as lacking [4,7]: 1) sufficient quality of evidence to inform practice; 2) comparative evidence between different splint deigns as related to efficacy, hand function, and occupational performance; and 3) patient-report about splint satisfaction and comfort (wearability). A review of 11 studies [7] implementing splints to manage TF included 2 that were level-1 evidence [10,32] as described Sackett [33], 5 were level-2 evidence [8,12,13,15,34], and 4 were level-3 evidence [6,11,16,35]. Of importance is that the same MCPJ blocking splint used in this RCT was also used in the level-1 studies [10,14]. In these studies, comparison of the MCPJ blocking splint was to finger-based splints that blocked motion at the PIPJ [14] or a DIPJ [10]. In this RCT, comparison is also made to finger-based RM splints that do not block joint motion but permit full finger mobility minus 20–25° of MCPJ motion.

A comparative study [20] between hand-based splints and finger-based RM splints implemented for protecting extensor tendon repairs concluded that the finger-based RM splints positively affected function, patient satisfaction, comfort and confirm non-comparative reports by others [17,36]. This RCT is different from other studies reviewed [7] as it includes patient-report of function and splint wearability. The importance of this inclusion is that patient perception has been linked to patient adherence [37]. Similarly, as noted by Tarbhai and colleagues [10] compared of MCPJ blocking splints to finger-based splints, our study also measures patient-report of function as assessed by the COPM.

The functionality of a RM splint over a hand-based splint has been evaluated with the Sollerman Test [20] and the photo-voice method [38]. For this RCT, the participants have been asked to describe their 6 weeks of splint wear experience via daily diaries. Generally, TF splint management studies [12,14,15] have used the DASH or quick-DASH to assess patient-report of function. The DASH has been included in this RCT so that comparison can be made with other studies that have used the DASH as an outcome measure.

The 2016 RM scoping review [19] informed the evidence that RM splints are being used clinically for a variety of purposes including TF despite the lack of evidence. This described scenario of practice application prior to substantiating evidence is not uncommon, thus confirming the need for this RCT.

A few strengths and limitations have been previously discussed, until this RCT is complete, there are a few additional points to consider-

- The sample size- although a power analysis was done, the participants in this study, may not fully represent the greater population of people with trigger finger.
- The risk of bias- given this is a clinical research study we did our best to minimize the risk of bias by using blinding methodology. However, at some point, the therapists, researcher, and participant will see the splint worn by each participant.
- The inclusion criteria did not exclude potential participants with co-morbidities such as carpal tunnel syndrome, diabetes mellitus, and rheumatoid arthritis. In our experience, these co-morbidities resemble the characteristics of patients with TF frequently managed by therapists.
- Patients with these aforementioned metabolic and inflammatory co-morbidities have a higher propensity to develop TF [32], which may indicate a more severe condition, which may subsequently be associated with worse health outcomes.
- Therapy contact of 3 therapy sessions was designed to reflect practice for which wearing the splint is the primary intervention. Different results may have been observed with more frequent therapy sessions and stricter controls; however, this would not have mirrored usual therapy practice.
- The Researchers of this study are relying on honest daily diary comments from participants, just as therapists do when interacting with patients in the clinical setting to evaluate the effectiveness of an intervention.

## Supporting information

S1 File SPIRIT 2013 checklist. (DOC)

S2 File Study protocol. Study protocol with information sheet, consent form, demographic questionnaire, and data collection form.
(PDF)

S3 File Database. (XLSX)

## References

[1] Akhtar S, Bradley MJ, Quinton DN, Burke FD. Management and referral for trigger finger/thumb. Bmj. 2005;331(7507):30–3. doi: 10.1136/bmj.331.7507.30 15994689 PMC558536

[2] Makkouk AH, Oetgen ME, Swigart CR, Dodds SD. Trigger finger: etiology, evaluation, and treatment. Curr Rev Musculoskelet Med. 2008;1(2):92–6. doi: 10.1007/s12178-007-9012-1 19468879 PMC2684207

[3] Langer D, Maeir A, Michailevich M, Luria S. Evaluating Hand Function in Clients with Trigger Finger. Occup Ther Int. 2017;2017:9539206. doi: 10.1155/2017/9539206 29097982 PMC5612741

[4] Lunsford D, Valdes K, Hengy S. Conservative management of trigger finger: A systematic review. J Hand Ther. 2019;32(2):212–21. doi: 10.1016/j.jht.2017.10.016 29290504

[5] Huisstede BM, Hoogvliet P, Coert JH, Fridén J. Multidisciplinary consensus guideline for managing trigger finger: results from the European HANDGUIDE Study. Phys Ther. 2014;94(10):1421–33. doi: 10.2522/ptj.20130135 24810861

[6] Evans RB, Hunter JM, Burkhalter WE. Conservative management of the trigger finger: a new approach. Journal of Hand Therapy. 1988;1(2):59–68.

[7] Leong LX, Chai SC, Howell JW, Hirth MJ. Orthotic intervention options to non-surgically manage adult and pediatric trigger finger: A systematic review. Journal of Hand Therapy. 2023;36(2):302–15. doi: 10.1016/j.jht.2023.05.016 37391318

[8] Valdes K. A retrospective review to determine the long-term efficacy of orthotic devices for trigger finger. J Hand Ther. 2012;25(1):89–95; quiz 6. doi: 10.1016/j.jht.2011.09.005 22265444

[9] Teo SH, Ng DCL, Wong YKY. Effectiveness of proximal interphalangeal joint–blocking orthosis vs metacarpophalangeal joint–blocking orthosis in trigger digit management: A randomized clinical trial. Journal of Hand Therapy. 2019;32(4):444–51. doi: 10.1016/j.jht.2018.02.007 30030005

[10] Tarbhai K, Hannah S, von Schroeder HP. Trigger finger treatment: a comparison of 2 splint designs. J Hand Surg Am. 2012;37(2):243–9, 9.e1. doi: 10.1016/j.jhsa.2011.10.038 22189188

[11] Patel MR, Bassini L. Trigger fingers and thumb: when to splint, inject, or operate. J Hand Surg Am. 1992;17(1):110–3. doi: 10.1016/0363-5023(92)90124-8 1538090

[12] Drijkoningen T, van Berckel M, Becker SJE, Ring DC, Mudgal CS. Night Splinting for Idiopathic Trigger Digits. Hand (N Y). 2018;13(5):558–62. doi: 10.1177/1558944717725374 28825334 PMC6109900

[13] Colbourn J, Heath N, Manary S, Pacifico D. Effectiveness of splinting for the treatment of trigger finger. J Hand Ther. 2008;21(4):336–43. doi: 10.1197/j.jht.2008.05.001 19006759

[14] Teo SH, Ng DCL, Wong YKY. Effectiveness of proximal interphalangeal joint-blocking orthosis vs metacarpophalangeal joint-blocking orthosis in trigger digit management: A randomized clinical trial. J Hand Ther. 2019;32(4):444–51. doi: 10.1016/j.jht.2018.02.007 30030005

[15] Pataradool K, Lertmahandpueti C. A proximal interphalangeal joint custom-made orthosis in trigger finger: Functional outcome. Hand Therapy. 2021;26(3):85–90. doi: 10.1177/17589983211018717 37904880 PMC10584048

[16] Rodgers JA, McCarthy JA, Tiedeman JJ. Functional distal interphalangeal joint splinting for trigger finger in laborers: a review and cadaver investigation. Orthopedics. 1998;21(3):305–9; discussion 9–10. doi: 10.3928/0147-7447-19980301-13 9547815

[17] Howell JW, Merritt WH, Robinson SJ. Immediate controlled active motion following zone 4–7 extensor tendon repair. J Hand Ther. 2005;18(2):182–90. doi: 10.1197/j.jht.2005.02.011 15891976

[18] Hirth M, Bennett K, Mah E, Farrow H, Cavallo A, Ritz M, et al. Early return to work and improved range of motion with modified relative motion splinting: A retrospective comparison with immobilization splinting for zones V and VI extensor tendon repairs. Hand Therapy. 2011;16:86–94.

[19] Hirth MJ, Howell JW, O’Brien L. Relative motion orthoses in the management of various hand conditions: A scoping review. J Hand Ther. 2016;29(4):405–32. doi: 10.1016/j.jht.2016.07.001 27793417

[20] Collocott SJF, Kelly E, Foster M, Myhr H, Wang A, Ellis RF. A randomized clinical trial comparing early active motion programs: Earlier hand function, TAM, and orthotic satisfaction with a relative motion extension program for zones V and VI extensor tendon repairs. J Hand Ther. 2020;33(1):13–24. doi: 10.1016/j.jht.2018.10.003 30905495

[21] Savage R, Pritchard MG, Thomas M, Newcombe RG. Differential splintage for flexor tendon rehabilitation: an experimental study of its effect on finger flexion strength. J Hand Surg Br. 2005;30(2):168–74. doi: 10.1016/j.jhsb.2004.10.014 15757770

[22] Savage R. The influence of wrist position on the minimum force required for active movement of the interphalangeal joints. J Hand Surg Br. 1988;13(3):262–8. doi: 10.1016/0266-7681_88_90082-4 3171289

[23] Sharma JV, Liang NJ, Owen JR, Wayne JS, Isaacs JE. Analysis of relative motion splint in the treatment of zone VI extensor tendon injuries. J Hand Surg Am. 2006;31(7):1118–22. doi: 10.1016/j.jhsa.2006.04.004 16945713

[24] Chung B, Chiu DTW, Thanik V. Relative Motion Flexion Splinting for Flexor Tendon Lacerations: Proof of Concept. HAND. 2019;14(2):193–6. doi: 10.1177/1558944717732063 28975818 PMC6436129

[25] Chan AW, Tetzlaff JM, Altman DG, Laupacis A, Gøtzsche PC, Krleža-Jerić K, et al. SPIRIT 2013 statement: defining standard protocol items for clinical trials. Ann Intern Med. 2013;158(3):200–7. doi: 10.7326/0003-4819-158-3-201302050-00583 23295957 PMC5114123

[26] Jackman M, Novak I, Lannin N. Effectiveness of hand splints in children with cerebral palsy: a systematic review with meta-analysis. Dev Med Child Neurol. 2014;56(2):138–47. doi: 10.1111/dmcn.12205 23848480

[27] Lalonde DH, Flewelling LA. Solving Hand/Finger Pain Problems with the Pencil Test and Relative Motion Splinting. Plast Reconstr Surg Glob Open. 2017;5(10):e1537. doi: 10.1097/GOX.0000000000001537 29184745 PMC5682181

[28] Howell JW, Ewald SG, Schwartz DA. Exercise relative motion orthoses: Use of the pencil test and variations of its use for assessing and managing different finger conditions. J Hand Ther. 2023;36(2):473–8. doi: 10.1016/j.jht.2022.10.004 36914489

[29] Finch E, Brooks D, Pw S, Mayo N. Physical Rehabilitation Outcome Measures2002.

[30] Hudak PL, Amadio PC, Bombardier C. Development of an upper extremity outcome measure: the DASH (disabilities of the arm, shoulder and hand) [corrected]. The Upper Extremity Collaborative Group (UECG). Am J Ind Med. 1996;29(6):602–8. doi: 10.1002/(SICI)1097-0274(199606)29:6&lt;602::AID-AJIM4&gt;3.0.CO;2-L 8773720

[31] Law M, Baptiste S, McColl M, Opzoomer A, Polatajko H, Pollock N. The Canadian occupational performance measure: an outcome measure for occupational therapy. Can J Occup Ther. 1990;57(2):82–7. doi: 10.1177/000841749005700207 10104738

[32] David M, Rangaraju M, Raine A. Acquired triggering of the fingers and thumb in adults. Bmj. 2017;359:j5285. doi: 10.1136/bmj.j5285 29191846

[33] Cook DJ, Guyatt GH, Laupacis A, Sackett DL. Rules of evidence and clinical recommendations on the use of antithrombotic agents. Chest. 1992;102(4 Suppl):305s-11s. 1395818

[34] Shiozawa R, Uchiyama S, Sugimoto Y, Ikegami S, Iwasaki N, Kato H. Comparison of splinting versus nonsplinting in the treatment of pediatric trigger finger. J Hand Surg Am. 2012;37(6):1211–6. doi: 10.1016/j.jhsa.2012.03.032 22624785

[35] Nemoto K, Nemoto T, Terada N, Amako M, Kawaguchi M. Splint therapy for trigger thumb and finger in children. J Hand Surg Br. 1996;21(3):416–8. doi: 10.1016/s0266-7681(05)80221-9 8771495

[36] Merritt WH. Relative motion splint: active motion after extensor tendon injury and repair. J Hand Surg Am. 2014;39(6):1187–94. doi: 10.1016/j.jhsa.2014.03.015 24862114

[37] Callinan NJ, Mathiowetz V. Soft versus hard resting hand splints in rheumatoid arthritis: pain relief, preference, and compliance. Am J Occup Ther. 1996;50(5):347–53. doi: 10.5014/ajot.50.5.347 8728664

[38] Cole T, Jamwal R, Hirth MJ. Photovoice to explore the patient experience of a relative motion orthosis following a hand injury. J Hand Ther. 2023;36(2):433–47. doi: 10.1016/j.jht.2023.02.001 37059599

