# Supplementary material for: Relative motion splints versus metacarpophalangeal joint blocking splints in the management of trigger finger: Study protocol for a randomized comparative trial
Source: PLoS One. 2024 Aug 13;19(8):e0307033. doi: 10.1371/journal.pone.0307033 (PMC11321552; doi:10.1371/journal.pone.0307033)
Supplement: S2 File — Study protocol with information sheet, consent form, demographic questionnaire, and data collection form. (PDF) [file pone.0307033.s002.pdf]

Study Protocol

A Randomized Comparative Trial of Relative Motion Splint Versus Metacarpophalangeal Joint Blocking Splint in the Management of Trigger Finger

Protocol Number:

Version 1.3, 07-04-2022

Name and Institution of Principle Investigator:

Leong Li Xian, Hospital Sultan Haji Ahmad Shah

Name and Institution of Co-Investigators:

Dr Chai Siaw Chui, Universiti Kebangsaan Malaysia

Dr Hanif Farhan Mohd Rasdi, Universiti Kebangsaan Malaysia

Dr Nur Rahimawati Abdul Rahman, Hospital Sultan Haji Ahmad Shah

Name and Institution of Expert Opinion:

Miss Julianne W. Howell, Self-Employed Consultant

Name and Address of Sponsor:

Nil

Study site:

Hospital Sultan Haji Ahmad Shah, Temerloh

CONTENTS

Topic	Page
List of Abbreviations	3
Research Synopsis	4
Background and Significance	5
Risk to Participants	20
Benefits to Participants	19
Risk Benefit Assessment	20
General Objective	11
Specific Objectives	11
Study Endpoints/Outcomes	11
Study Design and Methodology	11
Study Population	14
Sample Size	15
Inclusion Criteria	14
Exclusion Criteria	14
Withdrawal Criteria	14
Study Duration and Timeline	16
Study Visits and Procedures	16
Statistical Analysis Plan	19
Ethics of Study	20
Informed Consent/Assent Process	20
Privacy and Confidentiality	20
Conflict of Interest	21
Publication Policy	21
Termination of Study	21
References	22

ABBREVIATED FORM

A1	First Annular Pulley
COPM	Canadian Occupational Performance Measure
DASH	Disabilities of the Arm, Shoulder and Hand Outcome Measure
DIPJ	Distal Interphalangeal Joint
EDC	Extensor Digitorum Communis
EDM	Extensor Digiti Minimi
EIP	Extensor Indicis Proprius
FDP	Flexor Digitorum Profundus
FDS	Flexor Digitorum Superficialis
MCPJ	Metacarpophalangeal Joint
MCPJBG	Metacarpophalangeal Joint Blocking Group
PIPJ	Proximal Interphalangeal Joint
QuickDASH	Quick-Disabilities of the Arm, Shoulder and Hand Outcome Measure
RM	Relative Motion
RME Splint	Relative Motion Extension Splint
RMF Splint	Relative Motion Flexion Splint
RMG	Relative Motion Group
ICF	International Classification of Functioning, Disability and Health
IPJ	Interphalangeal Joint
SST	Stages of Stenosing Tenosynovitis
TF	Trigger Finger
VAS	Visual Analog Scale

RESEARCH SYNOPSIS

Study title	A Randomized Comparative Trial of Relative Motion Splint versus Metacarpophalangeal Joint Blocking Splint in the Management of Trigger Finger
Study Population	Patients who are diagnosed with TF involving the A1 pulley by a surgeon in the Orthopaedic Clinic, Hospital Sultan Haji Ahmad Shah, Temerloh, Pahang; satisfy the inclusion criteria; and referred for TF management at the Occupational Therapy Unit of this same public hospital during the period 1 January 2022 – 31 December 2024.
Study Design	A randomized comparative trial study. with random assignment of participants will be randomized assign into Relative Motion Group (RMG) that receives RM splint, or Metacarpophalangeal Joint Blocking Group (MCPJBG) that receives MCPJ blocking splint. Splint wear full time for 6 weeks
General Objective	To compare the effectiveness of RM and MCPJ blocking splints in terms of sign and symptoms, hand function, occupational performance, and perception of splint wearability (comfort and satisfaction) after six weeks of TF management.
Specific Objectives	1) To compare the effectiveness of RM splint and MCPJ blocking splint on TF severity after six weeks of TF management.<br>2) To compare the effectiveness of RM splint and MCPJ blocking splint on pain after six weeks of TF management.<br>3) To compare the effectiveness of RM splint and MCPJ blocking splint on finger triggering after six weeks of TF management.<br>4) To compare the effectiveness of RM splint and MCPJ blocking splint on hand function after six weeks of TF management.<br>5) To compare the effectiveness of RM splint and MCPJ blocking splint on occupational performance after six weeks of TF management.<br>6) To compare the effectiveness of RM splint and MCPJ blocking splint on comfort after six weeks of TF management.<br>7) To compare the effectiveness of RM splint and MCPJ blocking splint on satisfaction after six weeks of TF management.
Study endpoints/outcomes	1) There is a difference in terms of severity staging of TF.<br>2) There is a difference in terms of pain.<br>3) There is a difference in terms of finger triggering.<br>4) There is a difference in terms of hand function.<br>5) There is a difference in terms of occupational performance.<br>6) There is a difference in terms of comfort.<br>7) There is a difference in terms of satisfaction.
Sample Size	42 participants
Study Duration	1 January 2022 - 31 December 2024

1.0 BACKGROUND AND SIGNIFICANCE
1.1 BACKGROUND

Trigger finger (TF), or the stenosing tenosynovitis describes disproportionate size of a flexor tendon and tendon sheath at the first annular pulley (A1) at the metacarpophalangeal joint (MCPJ) (Lunsford et al. 2017). It is a common condition of the hand thought to be caused by inflammation or thickening of the tendon tissues that interferes with gliding of the tendon, which may in turn cause pain, clicking, catching and loss of motion (Makkouk et al. 2008). In the early stages, there may be local tenderness and swelling and with time a discrete nodule may form in the area of the A1 pulley (Gil et al. 2020).In severe cases, the affected finger can lock in flexion or extension and cause sharp pain when the finger is actively or passively unlocked. Although the cause of TF is unclear, there is a higher prevalence among people with metabolic diseases such as diabetes mellitus, hypothyroidism, mucopolysaccharidosis, and other inflammatory arthropathies (Gil et al. 2020). In adults the incidence of TF is 28:100,000 per year with a 2.6% lifetime incidence of TF in adults (Akhtar et al. 2005), and a higher prevalence in adult diabetics of between 5% and 20% (Kuczmarski et al. 2018). TF is reported to be more common in middle-aged individuals and woman are six times more likely to develop TF than men (Akhtar et al. 2005).

TF can affect hand functions which include precision of work, speed of hand performance, gross power grip, 3-jaw chuck pinch, and manipulation, causing difficulties in performing daily activities (Langer et al. 2017; Lunsford et al. 2017). Poor hand function in people with TF has been reported to contribute significantly to disability (Langer et al. 2016). Financially, treatment of TF by steroid injection or surgery is expensive as pointed out by Zhuang et al. (2020) who compared costs for two different health insurance payers in the United States of America. The cost of a single steroid injection was between $75.57 (commercial) and$49.26 (Medicare) and the cost of surgery was between $902 (commercial) and $853 (Medicare), respectively. Given that the success rate of a single steroid injection for TF was reported to be about 50%, half of all patients will either have a second injection or will have surgery recommended (Zhuang et al. 2020).

Hand therapy offers a non-invasive and easily accessed first line intervention option for management of TF. Usual therapy management consists of a custom-made splint, hand activity modification with or without nonsteroidal anti-inflammatory medications (Ryzewicz & Wolf 2006). As a result of a Delphi European consensus published in a 'Guideline for Managing Trigger Finger' (Huisstede et al. 2014) three interventions and the order of use were suggested based on the evidence available in 2014. The consensus was that the initial intervention be splint wear, the second intervention a corticosteroid injection, and the third, proceed to surgery. In addition, the level of intervention chosen must take into consideration the severity, duration, and previous intervention history of the individual's TF.

The primary purpose of a splint to manage TF is to limit flexor tendon excursion through the A1 pulley (Evans et al. 1988). To limit tendon excursion, various hand-based and finger-based splints have been described. These splints usually immobilize one finger joint, either the MCPJ, proximal interphalangeal joint (PIPJ), or distal interphalangeal joint (DIPJ). The MCPJ blocking splint appears to be the most frequently used (Colbourn et al. 2008; Drikoningen et al. 2017; Evans et al. 1988; Lindner-Tons & Ingell 1998; Patel & Bassini 1992; Tarbhai et al. 2012; Teo et al. 2018; Valdes et al. 2012). One publication immobilized the PIPJ (Teo et al. 2018) and two studies immobilized the DIPJ (Rodgers et al. 1998; Tarbhai et al. 2012). Tarbhai et al. (2012) compared a MCPJ blocking splint with a DIPJ blocking splint noted that the MCPJ blocking splint was preferred, with reasons cited as

being more stable, more comfortable, and less feeling of stiffness. Specifically, unlike DIPJ blocking splint that easily slipped off the finger, the MCPJ blocking splint allowed individuals with TF to perform tip-to-tip prehension which is important for hand function. The PIPJ blocking splint is the least studied splint for management of TF (Teo et al. 2018), which may be due to fabricate and fit issues.

Hand-based splints have been mainly advocated in the literature (Evans et al. 1988; Patel & Bassini 1992; Lindner-Tons & Ingell 1998). Evans et al. (1988) suggested a volar hand-based static splint with the MCPJ immobilized in neutral or 0° with the interphalangeal joints (IPJ) free. These authors hypothesized that wear of the TF splint would: 1) reduce the internal pressure about the A1 pulley; and 2) increase differential glide between the flexor tendons. In their study of 55 digits with TF, symptoms were resolved in 29 digits (52%) and symptoms improved in 12 digits (21%). Patel and Bassini (1992) immobilized 50 fingers in a hand-based splint that immobilized the affected finger's MCPJ in 10° to 15° of flexion leaving the IPJ freefor six weeks, with 33 fingers (66%) cured.

Lindner-Tons and Ingell (1998) referred to their hand-based MCPJ blocking splint as a minimal-profile splint design as it covers only the palmar aspect of the MCPJ of the involved finger and is secured by a ring around the finger's proximal phalanx. These authors projected that this splint would appeal to patients because it was less bulky, allowed for unrestricted hand function and cosmetically, and that these attributes might improve splint wear adherence and subsequently outcomes. Others have since adopted Lindner-Tons' splint design for their research (Colbourn et al. 2008; Drikoningen et al. 2017; Tarbhai et al. 2012; Teo et al. 2018; Valdes et al. 2012).

The relative motion (RM) splint is a finger-based design that holds potential for management of TF. The relative motion extension (RME) splint plus a wrist splint was originally used to manage extensor tendon repairs (Merritt 1998). Howell et al. (2005) (N=140) showed the RME splint to be protective, with no tendon ruptures and no active finger extension lags in 81% of the patients and allowed mobility as 79% of the patients had no loss of finger flexion. Because of the low profile, small size, and mobility afforded by the splint, patients were able to return to work in an average of 18 days post repair (Howell et al. 2005). In 2011, Hirth et al. had compared use of a RME splint without the wrist splint to management of zone V-VI extensor tendon repairs by immobilization for 4 weeks (Hirth et al, 2001). They too reported the benefits of the RME splint to be more cost effective, easy to fabricate, more convenient to wear, and allowed patients to return to work earlier than immobilization.

Historically the primary purpose of any splint designed to manage TF has been to block the MCPJ, PIPJ and/or DIPJ to reduce flexor tendon excursion, hence tendon/sheath irritation by the A1 pulley (Colbourn et al. 2008; Drijkoningen et al. 2017; Evans et al. 1988). The MCPJ blocking splint used in this study is intended to completely stop MCPJ motion and the RM splint to constrain MCPJ motion by at least 20-25°. It is unclear if the quadriga effect attributed to RM splint used after tenorrhaphy has a role in the treatment of TF. From biomechanical studies, we understand that the quadriga effect is levied by a RME splint to protect extensor tendons repairs in zone VI (Sharma et al. 2006) and by the RMF splint for flexor digitorum profundus (FDP) tendon repairs in zone III (Chung et al. 2017). The quadriga effect has been documented to be possible when multiple tendons are controlled by a single muscle, such as the extensor digitorum communis (EDC), FDP, and in the presence of tendinous interconnections of the extensor indicis proprius (EIP) and extensor digiti

minimi (EDM) to the EDC-multi-tendon unit (Henry et al. 2020; Hirth et al. 2011; Howell et al. 2005; Merritt 2014). As a result, when the affected finger's MCPJ is positioned in relatively greater extension or flexion, the active force is diverted more to the other tendons than the affected tendon (Merritt 2014). This principle of RM has been proven to protect repairs of the EDC, EIP, EDM in zones IV-VII and FDP in zones I-II (Henry et al. 2020; Hirth et al. 2011; Howell et al. 2005; Merritt 2014). It is unknown the role of quadriga in the management of other hand conditions such as sagittal band rupture (Catalano et al. 2006; Peelman et al. 2014), boutonniere deformity (Merritt & Jarrell 2020), digital nerve repair (Hirth et al. 2016) or undiagnosed hand pain (Lalonde 2013). Likewise, clinical experts reported use of the RM splint to manage TF (Hirth et al 2016). Yet, there is no evidence as to why the splint has worked.

For this study, we propose the following operational premises as to why a RM splint may work in the management of TF involving the A1 pulley. During controlled active motion: 1) the RME splint will lessen the passive tension of the extensor tendon(s) of the affected TF (quadriga), which biomechanically should lessen the force of flexion on the A1 pulley; 2) the RMF splint will levy the quadriga effect on the FDP to lessen the force generated and tendon excursion, and allow full FDS excursion (Savage 1988) and 3) the RME and RMF splints will decrease the amount of total active flexor tendon excursion by at least 25° without fully blocking MCPJ motion to support tendon healing (Evans et al. 1988).

The RM splint is a finger-based splint that is low profile, allows for a wide range of finger motion, permits unrestricted hand function with patients reporting greater satisfaction (Collocott et al. 2020; Hirth et al. 2016; Newington et al. 2021).TF may occur in one or multiple digits to which a RM splint can be adapted. Since TF involves primarily the flexor tendons, it is postulated that RM splint that positions the affected finger's MCPJ in relatively more flexion or extension will divert the tension of tendon to adjacent tendon and subsequently limit tendon excursion of the involved finger through A1 pulley. Since the RM splint reallocates the forces, but does not immobilize, protected active movement can be continued, and tendon healing supported. The design of the RM splint to be used when treating TF can be determined by first using the RM Pencil Test (Lalonde 2017) which simulates the RME or RMF position. The position that stops or reduces the patient's pain/triggering, determines which splint to fabricate and the degrees of difference between MCPJ to incorporate.

1.2 SIGNIFICANCE

Occupational therapy is a health profession that helps individuals to participate in their activities of everyday life (World Federation of Occupational Therapists 2012). Occupational therapists working in the field of hand and upper limb-related rehabilitation have placed a major concern on helping individuals with various conditions, including TF to optimize function and participation in all areas of their life. A review of literature regarding the role of splinting for TF revealed two areas lacking evidence: (1) attainments of hand function and occupational performance after splinting; and (2) patient's perception about splint wearability (specifically comfort and satisfaction).

Previous studies that used a splint to manage TF have been mostly focused on the resolution of symptoms as classified and measured by a variety of instruments. Typically, severity has been classified by the Stages of Stenosing Tenosynovitis (SST) proposed by Patel & Bassini (1992) and applied by Alsancak et al. (2015), Colborn et al. (2008), Evans et

al. (1998), Rodgers et al. (1998), and Valdes et al. (2012) as well as Green's (Wolfe et al. 2011) grade classification applied by Teo et al. (2018). The pain associated with TF has been measured by Visual Analog Scale (VAS) (Alsancak et al. 2015; Tarbhai et al. 2012; Valders et al. 2012) and Numerical Pain Rating Scale (NRPS) (Colborn et al. 2008; Drikoningen et al. 2017; Teo et al. 2018). Grip performance has been measured by a dynamometer (Colborn et al. 2008). Triggering has been measured by patient-report of frequency of triggering (Tarbhai et al. 2012) and the frequency of triggering during performance of ten active finger open and closes (Colborn et al. 2008). Studies that focused on hand function and occupational performance evaluation were limited with instruments used include only the Quick-Disabilities of the Arm, Shoulder and Hand Outcome Measure (QuickDASH) (Drikoningen et al. 2017; Teo et al. 2018) and the Canadian Occupational Performance Measure (COPM) (Tarbhai et al. 2012).

Limited use of hand function and occupational performance evaluation was reported by Lunsford at al. (2017) via their systematic review. They recommended using a validated self-reported outcome measure to evaluate functional capacity of individuals with TF in daily life. Overall, occupational therapists practice client-centred care that is not solely focused on patients' symptoms, but also their satisfaction with hand function and occupational performance. Brown and Chien (2010) noted that when patients are encouraged to express their problems and to participate in activities which are considered as more important and relevant to them, more important occupational issues can be identified.

Perceptions about splint wearability, especially comfort and satisfaction require attention as well. Out of 10 studies about splint for TF, only two studies had investigated the splint wearability (Drikoningen et al. 2017; Tarbhai et al. 2012). Tarbhai et al. (2012) compared 6 weeks of full time wear of MCPJ blocking splint to DIPJ blocking splint in two evenly matched cohorts. After six weeks of MCPJ blocking splint wear 10/13 digits were fully or partially improved compared to 7/15 digits managed with a DIPJ blocking splint. Initially before splint wear both TF groups reported no loss of hand function but after wearing their respective splints reported it was awkward to work. Additionally, after splint wear, patient-rated function for the MCPJ blocking splint was 'good' (n=7/13) and 'fair'(n=5/13), while 7/15 DIPJ blocking splint wearers rated equally as 'good' or fair'. More patients (n=10/13; 77%) rated the MCPJ blocking splint as comfortable than 9/15 (60%) patients in the DIPJ blocking splint group. Stiffness of the PIPJ was also a concern with those who wore the DIPJ blocking splint. The author's recommendation was to use the MCPJ blocking splint due to comfort and greater success rate. Although the MCPJ blocking splint showed better results than DIPJ blocking splint, the involved MCPJ was restricted thus, it makes holding objects difficult. Among the comments from participants about MCPJ blocking splint were: 1) awkward hand positions when working; 2) longer time was needed to do things; 3) edges of splint was digging. Drikoningen et al. (2017) had reported mean satisfaction of 5.8 points (SD + 3.3, 10 rating-scale) for six weeks night-wear MCPJ blocking splint. After six week of splint wear, 18 from 34 participants had received corticosteroid injection due to unsatisfied or noncompliance to the splint, or triggering was unable to resolve.

In contrast, the originators of the finger-based RME splint encouraged early use of the injured hand for light tasks such as eating, writing, self-care to prevent the secondary complications of adhesion formation and finger stiffness. Collocott et al. (2020) noted that extensor tendon repair individuals who were from RME group show better hand functions compare to controlled active motion group at four weeks for the Sollerman Hand Function

Test. Hirth et al.'s (2011) reported that manual workers such as carpenters and electricians were able to perform work tasks while wearing the RME splint. This is because the design of the RME splint allows enough finger flexion (minus between15-25°of MCPJ flexion of the involved finger) for holding a cup, writing, eating, or driving. With less restricted movement, individuals with TF may be able to return to work immediately and do work without interference from the RME splint.

A RM scoping review (Hirth et al.2016) suggested that RM splints were widely used for various purposes beyond the original intent which was to protect extensor tendon repairs. Specifically, RM splints have been used for sagittal band disruption, boutonniere deformity, digital nerve repair, PIPJ flexion stiffness and so on. The review highlighted those six RM experts, consisting of three consultants had initiated the RM concept nearly 40 years ago. Of them, three had more than ten years of clinical experience with RM splint. They were consulted regarding their use of both RME and RMF splints for other applications, one of which was management of TF. These experts suggested that use of a RM splint for patients with TF conditions effectively limited the amount of flexor tendon excursion at the A1 pulley, decreased PIPJ stiffness after surgical release of the A1 pulley for chronic TF, and lessened pain both after TF release and non-surgical management. From this review, further study aimed at the effectiveness of RM splints for non-surgical management of TF are needed to determine if RM splints have a role in the management of TF.

Given MCPJ blocking splint has wearability issues and RM splint has the potential to treat tendon-related condition, there is a need to evaluate the effectiveness of both splints in a more detailed and functional manner from various perspectives. By including symptoms (TF severity, pain, and number of triggering events), hand function, occupational performance, perception of splint wearability (comfort and satisfaction), we can develop a more comprehensive management of TF. Every single domain of the International Classification of Functioning, Disability and Health (ICF) model is included in this study (Figure 1.0), to form a big picture about the individual's health condition related to body function as well as to their function within society (World Health Organization [WHO]2002). From this study, we highlight the important of hand function and occupational performance as well as splint wearability. By doing this, we will be able to know the effectiveness of the splint and how it brings meaningful improvement to the individuals with TF.

TF causes difficulties in hand function and limitation of participation in work and activities of daily living. MCPJ blocking splint uses principle of immobilization by blocking MCPJ of the affected finger. Immobilization restricts the finger movement and reduces inflammation of the flexor tendon. In contrast, RM splint permits use of finger. Individual with TF can continue to work with minimal interruption of productivity due to the disability and the intervention. Splinting is a non-invasive and less costly intervention as compared to other treatment interventions such as steroid injection and surgery. Splinting does not give patient much financial pressure. It also reduces the economic burden of the country by minimizing the need to take medical leave and undergone further treatments. RM splint may also help to promote speedy recovery and reduce the impact on the quality of life due to patient's disability. Providing effective treatment can improve wellbeing of an individual and the community.

RM splint is a potential splint that we suggested to use for TF. There is no study about the effectiveness of RM splint for TF thus far. Since RM splint has a lot of benefits from the perspectives of its design and function, RM splint is potentially effective to improve TF and subsequently, become a better choice of splint for TF management. The change from an immobilization splint to a more functional splint is an evolution to the splint management for hand condition. Patient does not need to give up their hand function to avoid interference to the process of healing. For therapists, RM splint is easy to fabricate, cost effective, and time efficient. Data collected through this study have the potential to raise awareness of the importance of using function-focus approach rather than disability-focus approach as well as improve information provision for therapist and clinician in terms of management plan and intervention. Outcomes of this study will be beneficial to both therapist and patient because better management can be outlined to patient. Overall, it will give good impact to both the profession and patient.

2.0 RESEARCH QUESTION

Will there be any differences in terms of sign and symptoms, hand function, occupational performance, and perceptions about splint wearability between patients diagnosed with trigger finger (TF) involving the A1 pulley who are prescribed a relative motion (RM) splint for six weeks and those who received a metacarpophalangeal joint (MCPJ) blocking splint under the same conditions?

2.1 HYPOTHESES

Hypothesis 1: There is a difference in terms of severity staging as measured by Stages of Stenosing Tenosynovitis (SST) between patients diagnosed with TF involving the A1 pulley who are prescribed a RM splint for six weeks and those prescribed a MCPJ blocking splint under the same conditions.

Hypothesis 2: There is a difference in terms of pain as measured by Visual Analog Scale (VAS) between patients diagnosed with TF involving the A1 pulley who are prescribed a RM splint for six weeks and those prescribed a MCPJ blocking splint under the same conditions.

Hypothesis 3: There is a difference in terms of finger triggering as measured by number of triggering events in ten active fists between patients diagnosed with TF involving the A1 pulley who are prescribed a RM splint for six weeks and those prescribed a MCPJ blocking splint under the same conditions.

Hypothesis 4: There is a difference in terms of hand function as measured by Disabilities of the Arm, Shoulder and Hand (DASH) Outcome Measure between patients diagnosed with TF involving the A1 pulley who are prescribed a RM splint for six weeks and those prescribed a MCPJ blocking splint under the same conditions.

Hypothesis 5: There is a difference in terms of occupational performance as measured by Canadian Occupational Performance Measure (COPM) between patients diagnosed with TF involving the A1 pulley who are prescribed a RM splint for six weeks and those prescribed a MCPJ blocking splint under the same conditions.

Hypothesis 6: There is a difference in terms of comfort as measured by Visual Analog Scale (VAS) between patients diagnosed with TF involving the A1 pulley who are prescribed a RM splint for six weeks and those prescribed a MCPJ blocking splint under the same conditions.

Hypothesis 7: There is a difference in terms of satisfaction as measured by Visual Analog Scale (VAS) between patients diagnosed with TF involving the A1 pulley who are prescribed a RM splint for six weeks and those prescribed a MCPJ blocking splint under the same conditions.

2.2 RESEARCH QBJECTIVE

2.2.1 General Objective

To compare the effectiveness of RM and MCPJ blocking splints in terms of sign and symptoms, hand function, occupational performance, and perception of splint wearability (comfort and satisfaction) after six weeks of TF management.

2.2.2 Specific Objectives

Specific Objective 1: To compare the effectiveness of RM splint and MCPJ blocking splint on TF severity after six weeks of TF management.

Specific Objective 2: To compare the effectiveness of RM splint and MCPJ blocking splint on pain after six weeks of TF management.

Specific Objective 3: To compare the effectiveness of RM splint and MCPJ blocking splint on finger triggering after six weeks of TF management.

Specific Objective 4: To compare the effectiveness of RM splint and MCPJ blocking splint on hand function after six weeks of TF management.

Specific Objective 5: To compare the effectiveness of RM splint and MCPJ blocking splint on occupational performance after six weeks of TF management.

Specific Objective 6: To compare the effectiveness of RM splint and MCPJ blocking splint on comfort after six weeks of TF management.

Specific Objective 7: To compare the effectiveness of RM splint and MCPJ blocking splint on satisfaction after six weeks of TF management.

3.0 METHODOLOGY

3.1 Study Design

This research will use a randomized comparative trial design with random assignment of participants into Relative Motion Group (RMG) that receives RM splint, or Metacarpophalangeal Joint Blocking Group (MCPJBG) that receives MCPJ blocking splint.

3.1.1 Instrumentation

a. Demographic Questionnaire

This self-developed questionnaire will be used to collect the participant's personal information, including age, gender, occupation, hand dominance, finger involved, associated medical conditions, and duration of triggering.

b. Stages of Stenosing Tenosynovitis (SST)
In this study, the Stages of Stenosing Tenosynovitis (SST) will be used to measure the participant's severity staging of TF. This is a grading system that divides TF into six stages with each stage is theorized to describe a different mechanical problem. Typically, Stage 1 is normal; Stage 2 is uneven finger movement; Stage 3 is triggering or clicking or catching; Stage 4 is locking of finger in flexion or extension, which can be unlocked by active finger movement; Stage 5 is locking of finger in flexion or extension, which requires application of passive force to unlock; and Stage 6 is locked finger in flexion or extension (Patel & Bassini 1992).

c. Visual Analog Scale (VAS)
The Visual Analog Scale (VAS) will be used to measure the participant's pain as well as comfort and satisfaction with splint wear in this study. VAS is a 10cm line that can be scored in either centimetres or millimetres (Dale et al. 2011). For pain, the left end of the VAS will be labelled as "no pain" and the right end "extreme pain". For comfort, the left end will be labelled as "not at all comfortable' and the right end will be labelled as "extremely comfortable". For satisfaction, the left end will be labelled as "not at all satisfied" and the right end will be labelled as "extremely satisfied". At the times of evaluation, each patient will be asked to make a mark on the line to indicate his/her rating for pain, comfort or satisfaction. The distance of this mark from the left end of the line will be measured. Thong et al. (2018) had reported that VAS pain scale had excellent internal consistency (Pearson's =0.93) while Paungmali et al. (2012) reported that VAS had excellent test-retest reliability (Intraclass Correlation Coefficient, ICC=0.90). The minimal clinically important difference of the VAS pain scale is 12mm (Kelly 2001).

d. Number of Triggering Events in Ten Active Fists
Number of Triggering Events in Ten Active Fists will be used to measure the patient's triggering frequency (Finch et al. 2002). The number of triggering events (0 – 10) can be obtained by asking the patient to make 10 active full fists (Colbourn et al. 2008). Typically, this involves asking the patient to actively and fully open and close the fingers 10 times. If the finger persistently locks anytime during active finger opening and closing, a score of 10/10 will be assigned. If the patient has multiple TF per hand, he/she is only asked to open and close the fingers 10 times. The result is taken from the worst finger's performance. Time needed to make 10 active full fist will be recorded using a stopwatch or clock that reads in seconds.

e. Disabilities of the Arm, Shoulder and Hand (DASH) Outcome Measure
The Disabilities of the Arm, Shoulder and Hand (DASH) Outcome Measure will be used to measure hand function in this study. DASH is a 30-item, self-report questionnaire designed to assess the patient's health status during the previous week. The DASH consists of 21 items that assess performance of select activities; 5 items assess the severity of symptoms; and 4 items assess the impact of the problem on social function, work, sleep and self-image. Two optional modules specific to work, sports and/or performing arts will not be used in this study. The DASH has a total possible score of 0 to 100 with higher scores represent more severe disability or poorer hand function (Hudak et al. 1996; William 2014). The minimal clinically important difference for the DASH is 10.83 points (Franchignoni et al. 2014). Kleinlugtenbelt et al. (2018) reported excellent internal consistency (Cronbach's alpha=0.97) and excellent test-retest reliability (ICC=0.91) for the DASH.

f. Canadian Occupational Performance Measure (COPM)

The Canadian Occupational Performance Measure (COPM) will be used to measure the participant's occupational performance. COPM, developed by the Canadian Association of Occupational Therapist, in collaboration with Health and Welfare Canada (Law et al. 1990), is a widely known interview-based assessment tool for measuring occupational performance. COPM uses a client-centered approach and requires the patient to report the types of activities that he/she is unable to accomplish and later, prioritize the meaningfulness and importance of the selected activities. During the interview, the patient must report and rate the satisfaction and the performance of the activities in three occupational therapy areas, i.e., self-care, productivity, and leisure. The importance, satisfaction, and performance of an activity are rated using a 10-point rating scale. A benefit of the COPM is that self-perceived changes in occupational performance problems can be assessed over time (Law et al. 1990). Berardi et al. (2019) found that COPM has good construct validity (Cronbach's alpha=0.89) and excellent test-retest reliability for the performance subtest (ICC=0.99) and the satisfaction subtest (ICC = 0.98). The minimal clinically important difference of the COPM is 3.0 points and 3.2 points for performance subtest and satisfaction subtest, respectively (Tuntland et al.2016).

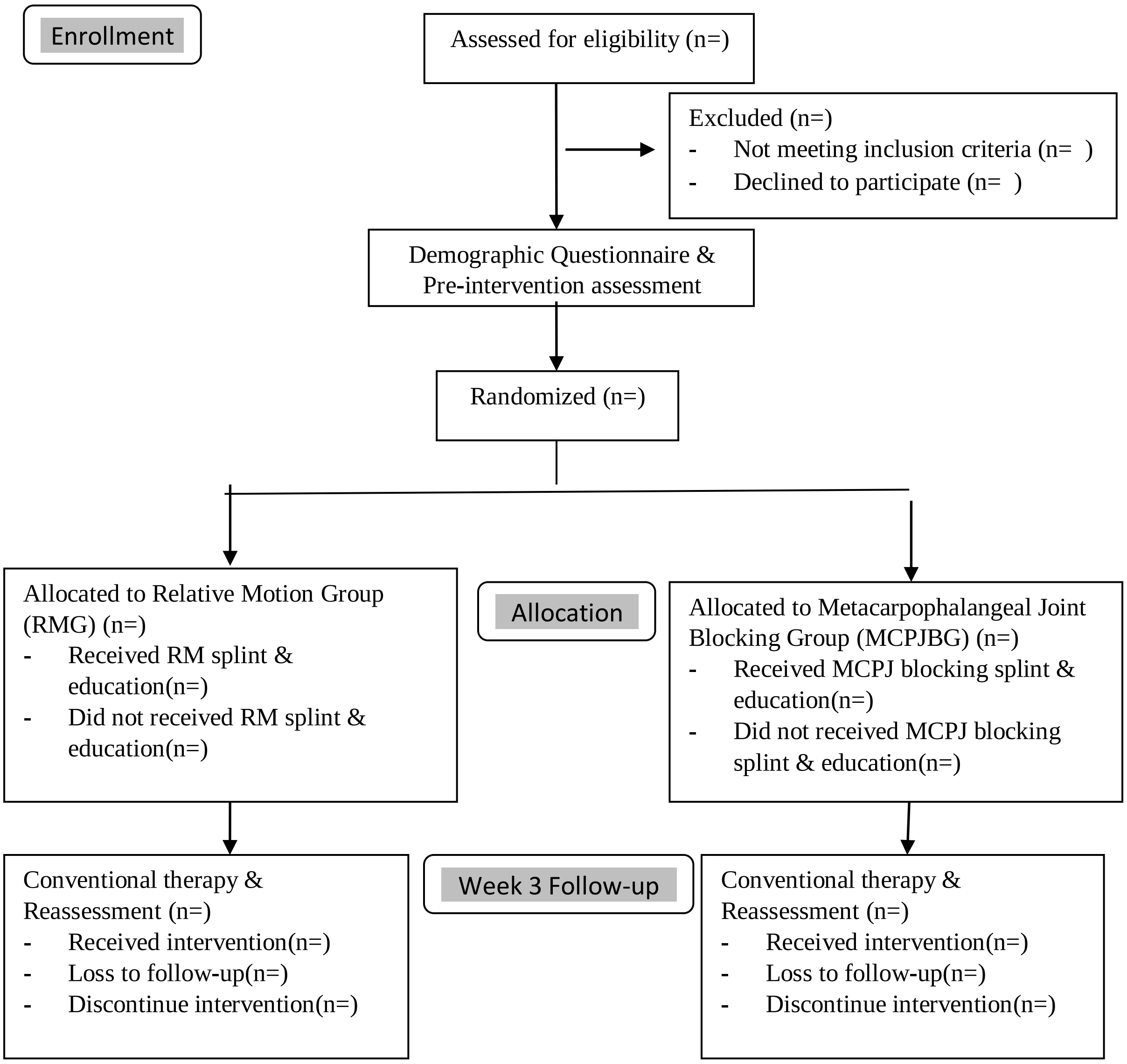

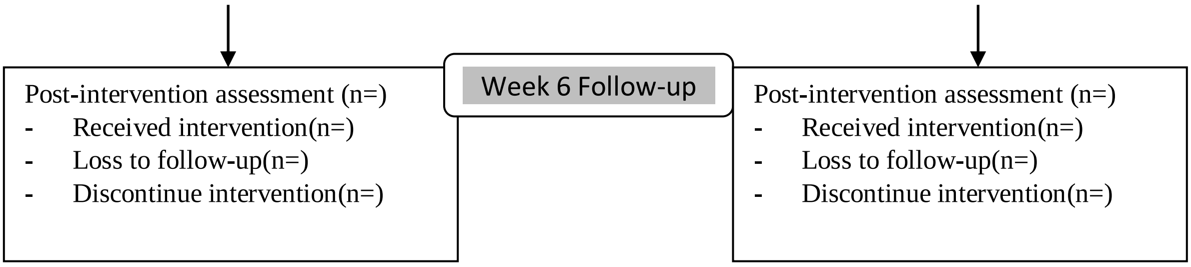

Figure 1.0 CONSORT flow chart. This figure shows the flow of patients through the trial according to the criteria recommended in the CONSORT Guidelines.

3.2 Study Sample

Patients who are diagnosed with TF involving the A1 pulley by a surgeon in the Orthopaedic Clinic, Hospital Sultan Haji Ahmad Shah, Temerloh, Pahang; satisfy the inclusion criteria; and referred for TF management at the Occupational Therapy Unit of this same public hospital during the period 1 January 2022 – 31 December 2024.

3.3 Inclusion Criteria

The inclusion criteria are:

- aged ≥ 21 years
- single or multiple TF
- unilateral or bilateral TF
- neutral MCPJ position can be attained during passive isolated MCPJ extension of the affected finger

3.4 Exclusion criteria

The exclusion criteria are:

- trigger thumb
- steroid injection of the affected finger within last six months
- A1 pulley release of the affected finger.
- history of fracture, tendon injury, nerve injury, Dupuytren's contracture, and other soft tissue injuries of the affected finger or adjacent fingers.

3.5 Withdrawal criteria

Participants can choose to withdraw at any time. Participants may be withdrawn if the investigator deems that it is detrimental or risky for the subject to continue. Withdrawn subjects will not be replaced by a new participant as we have already included the dropout rate (10%) into our sample size calculation.

3.6 Sample Size

A sampling of convenient method will be used to select eligible patients.

A prior TF research study (Teo et al. 2019) had reported that the result of pain score for pre-treatment = 4.65 (SD 2.39) and post-treatment = 3.40 (SD 2.44). G*Power was used to compute statistical power analyses. Cohen-d effect size is 0.5175152.

Given Cohen-d effect size needs to be converted into Cohen-f effect size in order to determine the sample size of this study for two ways ANOVA, therefore, after converting digitally, the Cohen-f effect size is 0.259.

This proposed study will need 32 participants when a Cohen-f effect size of 0.26, confident level of 95%, significant level of 5%, and statistical power of 80% is applied.

Since a 10% dropout rate is anticipated, a total of 36 participants will be needed for this study. Of this total, 18 patients will be allocated to the RM group (RMG) and 18 patients to the MCPJ blocking group (MCPJBG).

3.7 Study Duration and Timeline

Stage 1, review of article 12 months
Stage 2, data collection and data analysis 24 months
Stage 3, presentation and publication 12 months
Each patient will be involved in this study for 8 weeks. The duration of complete recruitment of 36 patients is estimated to be 2 years.

3.8 Study Visits and Procedures

This study will adopt a randomized comparative trial design. This study will involve the researcher, a counter staff, Therapist A, and Therapist B. The researcher will do the eligibility screening and splint fabrication. The counter staff will be involved in random assignment of participants to either the RMG or the MCPJBG. Therapist A, an occupational therapist with 10-years of experience is not a research team member and will conduct the pre-intervention assessments at Day-1 and the post-intervention assessments at Week-6. Therapist B, a senior occupational therapist with 20-years of experience also is not a research team member and will involve in conducting the conventional therapy program and assessment at Week-3. Due to the nature of study and the visibility of splint wearing (Jackman et al. 2013), the participants, the researcher, Therapist A, and Therapist B will not be blinded to the intervention.

Patients who have been recently diagnosed as having TF involving the A1 pulley and referred by the Orthopaedic Clinic, Hospital Sultan Haji Ahmad Shah to the hospital's Occupational Therapy Unit will make a new patient appointment for occupational therapy. Therapy will be provided at the Occupational Therapy Unit. Patients with TF will have a designated appointment and will be registered in the hospital information system as a new outpatient by the counter staff. The researcher will receive notice of a potential participant through the hospital information system on registration, and a preliminary screening of each potential patient will be done by the researcher. This screening will include review of the electronic referral letter and the past electronic medical records entered by the orthopaedic surgeon. After the preliminary screen of the records, the researcher will ask the potential participant additional questions according to inclusion and exclusion criteria to insure study eligibility.

Once study eligibility is confirmed, the researcher will provide the information sheet and explain the study to the potential patient. If the patient agrees to participate, he/she will then be asked to sign the informed consent form and assigned an Identity Number (ID) for anonymity purposes. After signing the informed consent form, the Therapist A will proceed to administer the Demographic Questionnaire and other pre-intervention (baseline) assessments i.e., SST, DASH, COPM, VAS (pain, comfort, and satisfaction), and the Number of Triggering Events in Ten Active Fists. After completing the pre-intervention assessments, the participant will be asked to go to a designated counter near the therapy room for simple random assignment to the RMG or MCPJBG. Assignment will be done via concealed allocation with selection of an envelope by the counter staff. The envelop is prepared by the researcher. The envelop will contain either a paper printed with A which indicates RMG and B which indicates MCPJBG. After the participant returning to the therapy room, he/she will give the group assignment paper to the researcher. The researcher will fabricate either the RM splint or MCPJ blocking splint based on the randomization of group allocation. Each type of splint, the RM splint and MCPJ blocking will be fabricated using a low temperature thermoplastic material with 3.2 mm thickness, 1% perforation, and 100% memory.

For participants assigned to RMG, either a RME splint or a RMF splint will be fabricated. The splint design will be dependent on the participant’s response to the RM pencil test carried out by the researcher. For this study, the RME pencil test will require the researcher to weave the pencil under the proximal phalanx of the affected finger(s) and over the dorsum of the adjacent fingers’ proximal phalanx. For the RMF pencil test the researcher will weave the pencil over the dorsum of the proximal phalanx of the affected finger(s) and under the proximal phalanx of the adjacent fingers. With the pencil in place, the participant will be asked to open and close their fingers several times. The participant will be asked to choose which pencil test, RME or RMF lessened their pain and/or triggering. If the answer is the RME position, then a RME splint will be prescribed. Conversely, if the answer is the RMF position, then a RMF splint will be prescribed.

For the RME splint, a strip of thermoplastic will be woven around on the proximal phalanx of all four fingers with the MCPJ of the affected finger(s) positioned in approximately 20°-25° more extension than MCPJ of the adjacent fingers. For the RMF splint, the thermoplastic strip will be woven with the MCPJ of the affected finger(s) positioned in approximately 20°-25° more flexion than the MCPJ of the adjacent fingers. The angle of MCPJ will be increased if 20°-25° flexion/extension is not enough to reduce/stop pain or triggering. For MCPJ blocking splint, the thermoplastic material will be moulded to contour the palm over the MCPJ and formed into a circumferential “ring” around the proximal phalanx of the affected finger(s). After the splint was fabricated, the participants will have to wear the splint for 15 minutes to check if the splint has pressure points that can cause pain or any redness of the skin. If there are pain and redness, the splint will be readjusted to prevent these pressure points. For all splints, each participant will be asked to assess the comfort and satisfaction with the splint before leaving the clinic.

The RMG and MCPJBG participants will receive same education and conventional treatment. Each participant will be instructed to wear the splint full time (24-hours) every day and night for six weeks, with the exception that the splint can be removed for hygiene purposes including washing hands and showering. No gripping when out of splint. To track splint wearing adherence, each participant will be given a diary to record the hours the splint was worn in a 24-hour period. Participant will also be asked to record what task(s)he/she has

removed the splint. Each participant will be informed about the condition of TF including symptoms, cause, risk factor, and treatment. Participants will also be encouraged to modify activities that may provoke their TF symptoms while wearing the splint. Suggestions for activity modification include stopping or limiting activities that involve repetitive hand use and prolonged gripping. Participants will be asked to contact the researcher if there are any problems with the splint that affect wear or to replace the splint if participant has accidentally lost their splint. The researcher will arrange an appointment as soon as possible with them to address these problems and make note of this interruption in intervention.

Each participant will be required to attend three sessions of therapy over the study period of six weeks. These sessions ideally will be the initial therapy session (Week-1), then three weeks later (Week-3), and six weeks after the initial session (Week-6). At Week-3, Therapist B will conduct the VAS assessment (comfort, and satisfaction) and provide the conventional therapy to each participant. Therapist B will perform a deep circular massage over A1 pulley for 5 minutes and each participant will be taught how to do the deep circular massage at home. The researcher will inspect each participant's splint and adjust it accordingly based on the participant's comments.

At Week-6, Therapist A will administer the post-intervention assessment for all participants. Table 1.0 outlines the procedure involved in RMG and MCPJBG. Figure 1.0 is the flowchart of the procedure.

Table 1.0 Procedure involved in Relative Motion Group and Metacarpophalangeal Joint Blocking Group.

**Relative Motion Group (RMG)**	**Metacarpophalangeal Joint Blocking Group (MCPJBG)**
**Week 1 (Day 1)**<br>Assessment<br>o Demographic questionnaire<br>o Pre-intervention assessments<br>- DASH<br>- COPM<br>- VAS (Pain, Comfort, & Satisfaction)<br>- Number of Triggering Events in Ten Active Fists<br>- SST<br><br>Splint<br>o RME pencil test<br>o RMF pencil test<br>o Fabricate RME splint or RMF splint<br>o MCPJ of the affected finger is approximately 20°-25° extension/flexion than the MCPJ of the adjacent fingers<br>o Increase the angle if 20°-25° MCPJ extension/flexion of affected finger is not enough to reduce/stop the triggering/pain<br><br>Patient Education<br>o Splint wear full time for 6 weeks, only remove for hygiene purpose<br>o No gripping when out of splint<br>o Provide a diary to record everyday splint wear duration (hours)<br>o Record what task the splint is removed for<br>o About TF condition	**Week 1 (Day 1)**<br>Assessment<br>o Demographic questionnaire<br>o Pre-intervention assessments<br>- DASH<br>- COPM<br>- VAS (Pain, Comfort, & Satisfaction)<br>- Number of Triggering Events in Ten Active Fists<br>- SST<br><br>Splint<br>o Fabricate MCPJ blocking splint<br>o MCPJ positioned at 0°<br><br>Patient Education<br>o Splint wear full time for 6 weeks, only remove for hygiene purpose<br>o No gripping when out of splint<br>o Provide a diary to record everyday splint wear duration (hours)<br>o Record what task the splint is removed for<br>o About TF condition<br>o Reduce or avoid activities such as repetitive hand use and prolonged grip or any action that causes pain/triggering

○ Reduce or avoid activities such as repetitive hand use and prolonged grip or any action that causes pain/triggering	
**Week 3**<br>Reassessment<br>○ VAS (Comfort & Satisfaction)<br><br>Conventional Therapy<br>○ Deep circular massage over A1 pulley for 5 minutes<br>○ Review and adjust splint	**Week 3**<br>Reassessment<br>○ VAS (Comfort & Satisfaction)<br><br>Conventional Therapy<br>○ Deep circular massage over A1 pulley for 5 minutes<br>○ Review and adjust splint
**Week 6**<br>Reassessment<br>○ Post-intervention assessments<br>- DASH<br>- COPM<br>- VAS (Pain, Comfort, & Satisfaction)<br>- Number of Triggering Events in Ten Active Fists<br>- SST	**Week 6**<br>Reassessment<br>○ Post-intervention assessments<br>- DASH<br>- COPM<br>- VAS (Pain, Comfort, & Satisfaction)<br>- Number of Triggering Events in Ten Active Fists<br>- SST

3.9 Statistical Analysis Plan

Descriptive statistics will be used to summarize the Demographic data including age, gender, occupation, hand dominance, digit involved, associated medical conditions, and duration of triggering. Data will be analysed and described in total numbers, mean, and the pattern of data. Descriptive statistics will also be used to demonstrate the assessment outcomes including DASH, COPM, VAS (pain, comfort, and satisfaction), Number of Triggering in Ten Active Fists, and SST. An independent sample *t*-test, set at the significant level of 0.05 will be used to compare the baseline data between RMG and MCPJBG.A Two Way Mixed-ANOVA, set at the significant level of 0.05will be used to compare the assessment outcomes, including DASH, COPM, VAS (pain, comfort, and satisfaction), Number of Triggering in Ten Active Fists, and SST between the RMG and MCPJBG. The interaction effect between these different variables will be analysed too. The results will show the percentage of variance in each effect regardless of size (Hypotheses 1 – 7).

3.10 Risk and Benefit to Study Participants

Benefit:
Splint is an evidenced-based treatment for TF management. For the past three decades, splint was studied and applied by clinician as a management of TF (Colborn et al. 2008; Drikoningen et al. 2017; Evans et al. 1998; Patel & Bassini 1992; Rodgers et al. 1998; Tarbhai et al. 2012; Valdes et al. 2012). It is safe to be applied to patients with TF. Based on the published studies, the success rate of splinting on TF ranged from 47% to 93% (Lunsford et al. 2017).

Risk:
No serious adverse event was reported in the published studies. Two out of 30 patients reported having grade I pressure sore (painful redness of skin without loss of skin dermis), but the pressure sore healed without further progression (Pataradool and Lertmahandpueti 2021). The risk of apply a splint as a treatment to patients with TF is minimal.

3.11 Risk Benefit Assessment

As discussed above, splint is a treatment option for TF for more than 20 years. With minimal risk, a new design of splint may give better benefit to both the therapist and the patient in terms of time spent, costing, and comfortability.

3.12 Adverse Event

Wear of the splint may cause pressure injury to the skin. The researcher will take necessary precaution and careful supervision throughout the entire process of splint fabrication to minimise the potential of causing pressure injury. After the splint was fabricated, the participants will have to wear the splint for 15 minutes to check if the splint has pressure points that can cause pain or any redness of the skin. If there are pain and redness, the splint will be readjusted to prevent these pressure points. At week-3 and week-6, the comfort of splint will be assessed. The researcher will record if there is any pressure injury. The participants also will be advised to report immediately (contact by phone or text) if there is sign of pressure injury to the skin.

The experimental group will receive RM splint while the comparator group will receive MCPJ blocking splint. Both group in the study will receive intervention to prevent the comparator group from receiving potential harms of non-treatment. Safety issue of patients will be constantly monitored by researcher during the period of study. Participants will be asked to contact the researcher immediately if there are any problems arise.

3.13 Ethical of Study

This randomized comparative trial will apply for ethics approval from the Medical Research and Ethics Committee of Ministry of Health. Prior enrolling into this study, patients will be given an information sheet that describes the purpose and design of the study to read. Patients will be required to sign the informed consent form if they agreed to participate. This study will be registered at Clinical Trials Registry.

3.14 Informed Consent

During the first visit to Occupational Therapy Unit, the patient will be screened based on inclusion and exclusion criteria. Once study eligibility is confirmed, the researcher will provide the information sheet and explain the study to the potential patient. If the patient agrees to participate, he/she will then be asked to sign and date the informed consent form.

3.15 Privacy and Confidentiality

Participant's names will be kept on a password-protected database and will be linked only with a study identification number for this research. The identification number instead of patient identifiers will be used on subject data sheets. The data from this study will be made into a report which may be published. Once the data have been gathered and the research completed, the raw materials will be kept in the locked file cabinet in the office of the researcher. Electronic data will be securely kept using password. Access to the data is only by the researcher. After 5 years, the material will be shredded and discarded. Participants will not be allowed to view their personal study data, as the data will be consolidated into a database. Participants can write to the researcher to request access to study findings.

3.16 Conflict of Interest

The researchers declare they have no conflict of interest.

3.17 Publication Policy

No personal information will be disclosed and participants will not be identified when the findings of the study are published. Permission from the Director General of Health, Malaysia will be obtained prior to publication.

3.18 Termination of Study

The researchers may decide to terminate the study at any time. Participants will be informed if the study is terminated and follow-up visits will be arranged if needed. Reasons that may require termination of the study include:

- It becomes apparent that patient enrolment is unsatisfactory with respect to quality or quantity.
- Date and record are inaccurate and/or incomplete.
- Deliberate violation of the protocol.
- The incidence and/or severity of adverse events in these studies indicate a potential health hazard caused by the treatments under trial.

REFERENCES

1. Akhtar, S., Bradley, M.J., Quinton, D.N. & Burke, F.D. 2005. Management and referral for trigger finger/thumb. *British Medical Journal* 331:30-33
2. Alsancak, S., Güner, S. & Bilgin, S. 2015. Efficacy of splinting variations in two different treatment protocols in trigger finger. *Journal of Prosthetics and Orthotics* 27(1):17-22
3. Aneesha Sudhir Kopare, Vidhi Shah, M Vijaya Kumar, Mohammed Ubaid Khan, Diksha Gondkar& Tushar J Palekar. 2019. Effect of hand grip web exercises and theraputty exercises on manual dexterity in geriatric population. *International Journal of Basic and Applied Research* ISSN 2249-3352 (P) 2278-0505 (E)
4. Aneesha Sudhir Kopare, Vidhi Shah, M Vijaya Kumar, Mohammed Ubaid Khan, Diksha Gondkar, Tushar J Palekar. 2019. Effect of hand grip web exercises and theraputty exercises on manual dexterity in geriatric population. *International Journal of Basic and Applied Research* ISSN 2249-3352 (P) 2278-0505 (E)
5. Berardi, A., Galeoto, G., Guarino, D., Marquez, M.A., Santis, R.D., Valente, D., Caporale, G. & Tofani, M. 2019. Construct validity, test-retest reliability, and the ability to detect change of the Canadian Occupational Performance Measure in a spinal cord injury population. *International Spinal Cord* 5(52):1-8
6. Brown, T. & Chien, W.C.W. 2010. Top-down or bottom-up occupational therapy assessment: Which way do we go? *British Journal of Occupational Therapy* 73(3):95
7. Catalano, L.W., Gupta, S., Ragland, R., Glickel, S.Z., Johnson, C. & Barron, A. 2006. Closed treatment of nonrheumatoid extensor tendon dislocations at the metacarpophalangeal joint. *The Journal of Hand Surgery* 31A:242-245
8. Chung, B., Chiu, D.T.W. &Thanik, V. 2019. Relative motion flexion splinting for flexor tendon lacerations: proof of concept. *Hand (N.Y.)* 14(2):193-196
9. Colbourn, J., Heatth, N., Manary, S. & Pacifico, D. 2008. Effectiveness of splinting for the treatment of trigger finger. *Journal of Hand Therapy* 21(4):336-343
10. Collocott, S.J.F., Kelly, E., Foster, M., Myhr, H., Wang, A. & Ellis, R.F. 2020. A randomized clinical trial comparing early active motion programs: Earlier hand function, TAM, and orthotic satisfaction with a relative motion extension program for zones V and VI extensor tendon repairs. *Journal of Hand Therapy* 33:13-24
11. Drijkoningen, T., Berckel, M.V., Becker, S.J.E., Ring, D.C. & Mudgal C.S. 2018. Night splint for idiopathic trigger finger. *Hand (N. Y.)* 13(5):558-562
12. Evans, R.B., Hunter, J.M. & Burkhalter, W.E. 1988. Conservative management of the trigger finger: A new approach. *Journal of Hand Therapy*59-68
13. Finch, E., Brooks, D., Stratford, P. & Mayo, N. 2002. *Physical Rehabilitation Outcome Measures: A Guide to Enhanced Clinical Decision Making*. 2nd ed. Hamilton, Ontario: BC Decker Inc.
14. Franchignoni, F., Vercelli, S., Giordano, A., Sartorio, F., Bravini, E. & Ferriero, G. 2014. Minimal clinically important difference of the Disabilities of the Arm, Shoulder and Hand Outcome Measure (DASH) and its shortened version (QuickDASH). *Journal of Orthopaedic and Sports Physical Therapy* 44(1):30-39
15. Gabriella Iohom. 2006. Chapter 11 – Clinical assessment of postoperative pain. *Postoperative Pain Management: An Evidence-Based Guide to Practice*. 102-108
16. Gil, J.A., Hresko, A.M. & Weiss, A.P.C. 2020. Current concepts in the management of trigger finger in adults. *Journal of Am Academic Orthopaedic Surgery* 28:e642-e650
17. Halfaker, D.A., Akeson, S.T., Hathcock, D.R., Mattson, C. & Wunderlich, T.L. 2011. *Pain Procedures in Clinical Practice - Third Edition*. 13-22

18. Hamilton, G.F., McDonald, C. & Chenier, T.C. 1992. Measurement of grip strength: Validity and reliability of the sphygmomamometer and Jamar grip dynamometer. *Journal of Orthopaedic & Sports Physical Therapy* 16(5);215-219
19. Henry, S.L. & Howell, J.W. 2020. Use of a relative motion flexion orthosis for postoperative management of zone I/II flexor digitorum profundus repair: a retrospective consecutive case series. *Journal of Hand Therapy*33(3):296-304
20. Hirth, M.J., Bennett, K., Mah, E., Farrow,H.C.,Cavallo,A.V.,Ritz, M. & Findlay, M.W. 2011. Early return to work and improved range of motion with modified relative motion splinting: A retrospective comparison with immobilization splinting for zones V and VI extensor tendon repairs. *Journal of Hand Therapy* 16:86-94
21. Hirth, M.J., Howell, J.W. & O'Brien, L. 2016.Relative motion orthoses in the management of various hand conditions: A scoping review. *Journal of Hand Therapy* 1-27
22. Hirth, M.J., Howell, J.W. & O'Brien, L. 2017. Two case reports – Use of relative motion orthoses to manage extensor tendon zones iii and iv and sagittal band injuries in adjacent fingers. *Journal of Hand Therapy* 30:546-547
23. Howell, J.W., Merritt, W.H. & Robinson, S.J. 2005. Immediate controlled active motion following zone 4-7 extensor tendon repair. *Journal of Hand Therapy* 18:182-190
24. Hudak, P.L., Amadio, P.C., Bombardier, C. & the Upper Extremity Collaborative Group (UECG). 1996. Development of an upper extremity outcome measure: The DASH (Disabilities of the arm, shoulder, and head). *American Journal of Industrial Medicine* 29:602-608
25. Huisstede, B.M., Hoogvliet, P., Coert, J.H. &Fridén, J. 2014. Multidisciplinary consensus guideline for managing trigger finger: Results from the European HANDGUIDE Study. *Journal of the American Physical Therapy Association* 94:1421-1433
26. Jackman, M., Novak, I. & Lannin, N. 2013. Effectiveness of hand splints in children with cerebral palsy: a systematic review with meta-analysis. *Developmental Medicine & Child Neurology* 56:138-147
27. Kelly, A.M. 2001. The minimum clinically significant difference in visual analogue scale pain score does not differ with severity of pain. *Emergency Medicine Journal* 18(3):205-207
28. Kleinlugtenbelt, Y.V., Krol, R.G., Bhandari, M. Goslings, J.C., Poolman, R.W., Scholtes, V.A.B. 2018. Are the Patient-Rated Wrist Evaluation (PRWE) and the Disabilities of the Arm, Shoulder and Hand (DASH) Questionnaire use in distal radial fractures truly valid and reliable? *Bone and Joint Research*7(1):36-45
29. Kuczmarski, A.S., Harris, A.P., Gil, J.A. & Weiss, A.P.C. 2019. Management of diabetic trigger finger. *Journal of Hand Surgery Am* 44(2):150-153
30. Lalonde, D.H. 2013. ASHT invited speech – How the wideawake approach is changing hand surgery and hand therapy: Inaugural AAHS sponsored lecture at the ASHT Meeting, San Diego, 2012. *Journal of Hand Therapy* 26(2):175-178
31. Lalonde, D.H. 2017. Solving hand/finger pain problems with the pencil test and relative motion splint. *Plastic and Reconstructive Surgery – Global Open* Operative technique 1-2
32. Langer, D., Maeir, A., Michailevich, M. & Luria, S. 2017. Evaluating hand function in clients with trigger finger. *Hindawi Occupational Therapy International* 1-8
33. Langer, D., Maeir, A., Michailevich, M., Applebaum, Y. & Luria, S. 2016. Using the international classification of functioning to examine the impact of trigger finger. *Disability and Rehabilitation* 38(26):2530-2537

34. Law, M., Baptiste, S., McColl, M.A., Opzoomer, A., Polatajko, H.J. & Pollock, N. 1990. The Canadian Occupational Performance Measure: An outcome measure for occupational therapy. *Canadian Journal of Occupational Therapy* 57(2);82-87
35. Lindner-Tons, S. & Ingell, K. 1998. An alternative splint design for trigger finger. *Journal of Hand Therapy* 11:206-208
36. Lunsford, D., Valdes, K. & Hengy, S. 2017. Conservative management of trigger finger: A systematic review. *Journal of Hand Therapy*1-9
37. Macdermid, J.C., Fehr L. & Lindsay, K. 2002. The Effect of physical factors on grip strength and dexterity. *The British Journal of Hand Therapy* 7(4);112-118
38. Makkouk, A.H., Oetgen, M.E., Swigart, C.R. & Dodds, S.D. 2008. Trigger finger: etiology, evaluation, and treatment. *Current Reviews in Musculoskeletal Medicine* 1:92-96
39. Merritt, W. H. 1998. Written on behalf of the stiff finger. *Journal of Hand Therapy* 11(2):74-79
40. Merritt, W.H. 2014. Relative motion splint: Active motion after extensor tendon injury and repair. *Journal of Hand Surgery* 39(6):1187-1194
41. Merritt, W.H. & Jarrell, K. 2020. A paradigm shift in managing acute and chronic boutonniere deformity: Anatomic rationale and early clinical results for the relative motion concept permitting immediate active motion and hand use. *Annals of Plastic Surgery* 84(2):S142-S150
42. Merritt, W.H., Wong, A.L. & Lalonde, D.H. 2020. Recent developments are changing extensor tendon management. *Plastic and Reconstructive Surgery* 145:617e
43. Muir, S.W., Corea, C.L. &Beaupre, L. 2010. Evaluating change in clinical status: Reliability and measures of agreement for the assessment of glenohumeral range of motion. *North American Journal of Sports Physical Therapy* 5(3):98-110
44. Pataradool, K. & Lertmahandpueti, C. 2021. A proximal interphalangeal joint custom-made orthosis in trigger finger: Functional outcome. *Hand Therapy* 26(3):85-90
45. Patel, M.R. & Bassini, L. 1992. Trigger fingers and thumb: When to splint, inject, or operate. *Journal of Hand Surgery* 17A:110-113
46. Peelman, J., Markiewitz, A., Kiefhaber, T. &Stern, P. 2014. Splintage in the treatment of sagittal band incompetence and extensor tendon subluxation. *Journal of Hand Surgery* 40(3):287-290
47. Paungmmali, A., Sitilertpisan, P., Taneyhill, K., Pirunsan, U. & Uthaikhup, S. 2012. Intrarater reliability of pain intensity, tissue blood flow, thermal pain threshold, pressure pain threshold and lumbo-pelvic stability tests in subjects with low back pain. *Asian Journal of Sports Medicine* 3(1):8-14
48. Riddle, D.L., Rothstein, J.M. & Lamb, R.L. 1987. Goniometric reliability in a clinical setting. Shoulder measurements. *Physical Therapy* 67(5);668-673
49. Rodgers, J.A., McCarthy, J.A. & Tiedeman, J.J. 1998. Functional distal interphalangeal joint splinting for trigger finger in laborers: a review and cadaver investigation. *Orthopedics* 21(3):305-309
50. Rose, B.W., Kasch, M.C., Aaron, D.H. & Stegink-Jansen, C.W. 2011. Does hand therapy literature incorporate the holistic view of health and function promoted by the world health organization? *Journal of Hand Therapy*24:84-88
51. Ryzewicz, M. & Wolf, J.M. 2006. Trigger digits: Principles, management, and complications. *Journal of Hand Surgery* 31A:135-146
52. Savage, R. 1988. The influence of wrist position on the minimum force required for active movement of the interphalangeal joints. *Journal of Hand Surgery* 13(3):262-268

53. Serap, A., Gűner, S., & Bilgin, S. 2015. Efficacy of splinting variations in two different protocols in trigger thumb. *Journal of Prosthetists and Orthotists* 27:17-22
54. Sharma, J.V., Liang, N.J., Owen, J.R., Wayne, J.S. & Issacs, J.E. 2006. Analysis of relative motion splint in the treatment of zone VI extensor tendon injuries. *Journal of Hand Surgery* 31A:1118-1122
55. Steiner, W.A., Ryser, L., Huber, E., Uebelhart, D., Aeshilimann, A., & Stucki, G. 2002. Use of the ICF model as a clinical problem-solving tool in physical therapy and rehabilitation medicine. *Physical Therapy* 82(11):1095-1107
56. Tarbhai, K., Hannah S. & Schroeder, H.P.V. 2012. Trigger finger treatment: A comparison of 2 splint designs. *Journal of Hand Surgery* 3(2):243-249
57. Teo, S.H., Chen, D.L.N. & Wong, Y.K.Y. 2018. Effectiveness of proximal interphalangeal joint-blocking orthosis vs metacarpophalangeal joint-blocking orthosis in trigger digit management: A randomized clinical trial. *Journal of Hand Therapy* 32(2019);444-451
58. Thong, I.S.K., Jensen, M.P., Miro, J. & Tan, G. 2018. The validity of pain intensity measures: what do the NRS, VAS, NRS, and FPS-R measure? *Scandinavian Journal of Pain* 18(1):99-107
59. Tuntland, H., Aaslund, M.k., Langeland, E., Espehaug, B., & Kjeken, I. 2016. Psychometric properties of the Canadian Occupational Performance Measure in home-dwelling older adults. *Journal of Multidisciplinary Healthcare* 9:411-423
60. Valdes, K. 2012. A retrospective review to determine the long-term efficacy of orthotic devices for trigger finger. *Journal of Hand Therapy* 25:89-96
61. Williams, N. 2014. Questionnaire review: DASH. *Occupational Medicine* 64:67-68
62. Wolfe, S., Pederson, W. Kozin, S., & Cohen, M. 2011. Tendinopathy. *Green's Operative Hand Surgery, 6th ed. London: Churchill Livingstone* 2071-3072
63. World Federation of Occupational Therapists. 2010. Client-Centredness in Occupational Therapy.
64. World Health Organization. 2001. International Classification of Functioning, Disability and Health. 2001:29-30
65. World Health Organization. International Classification of Functioning, Disability and Health. 2001:29-30
66. Zhuang, T., Wong, S., Aoki, R., Zeng, E., Ku, S., & Kamal, R.N. 2020. A cost-effectiveness analysis of corticosteroid injections and open surgical release for trigger finger. *Journal of Hand Surgery Am* 45(7):597-609

PATIENT INFORMATION SHEET AND INFORMED CONSENT FORM

1. Research title:

A Randomized Comparative Trial of Relative Motion Splint Versus Metacarpophalangeal Joint Blocking Splint in the Management of Trigger Finger.

2. Name of investigator and institution:

Leong Li Xian, Hospital Sultan Haji Ahmad Shah

3. Introduction:

This is an Occupational Therapist study that does not involve medical treatment or administration of invasive procedure to participant by a medical doctor. You are invited to participate in an experimental research study because you have diagnosed with trigger finger that requires Occupational Therapy. Trigger finger can be described as a discrepancy of size between the flexor tendon and tendon sheath. It can cause local swelling, finger pain and finger triggering. In severe cases, the affected finger can lock in flexion or extension. Occupational therapy for patients with trigger finger includes a custom-splint, guided exercises and massage (conventional therapy), and education in cause and prevention. Specific details of the study are described in this document. It is important that you understand why the study is being done and what it will involve. Please take your time to read through and consider this information carefully before you decide if you are willing to participate. Please ask the researcher if you are unclear about anything or if you like more information. Once you are satisfied that you understand what this study entails, and that you wish to participate, you must sign this informed consent form.

Your participation in this study is voluntary. You do not have to be in this study if you do not want to. You may also refuse to answer any questions you do not want to answer. If you volunteer to be in this study, you may withdraw from it at any time. If you withdraw, any data collected from you up to your withdrawal will still be used for the study. Your refusal to participate or withdrawal will not affect any medical or health benefits to which you are otherwise entitled.

This study has been approved by the Medical Research and Ethics Committee, Ministry of Heath Malaysia.

4. What is the purpose of the study?

The purpose of this study is to compare the Relative Motion (RM) Splint and Metacarpophalangeal Joint (MCPJ) Blocking Splint in the management of trigger finger. We want to see which splint is better in reduce the pain and triggering of the affected finger, increase the hand function and allow the participants to play their roles in daily lives. We also want to study which splint can provide better experience during six weeks of splint wearing.

A total of 42 participants like you will be participating in this study. The whole study will last about 3 years; however, your participation will be for about 6 weeks.

5. What kind of therapy will I receive?

If you agree to participate in the study, you will be asked to complete a series of questionnaires and complete a few measurements. You will then be randomly assigned to one of the treatment groups below by drawing from concealed envelopes. You have equal chance of being assigned to each of the groups.

Group 1: Participants will be prescribed with RM splint.
Group 2: Participants will be prescribed with MCPJ blocking splint.

Regardless of your group allocation, you will need to complete the following questionnaires and measurements administered by the therapist at the beginning of the hand therapy treatment (pre-intervention/baseline assessment) and after 6 weeks of hand therapy treatment (post-intervention):

1) Demographic Questionnaire
2) Stages of Stenosing Tenosynovitis
3) Visual Analog Scales
4) Number of Triggering Events in Ten Active Fists
5) Disabilities of the Arm, Shoulder and Hand (DASH) Outcome Measure
6) Canadian Occupational Performance Measure (COPM)

6. What are the responsibilities when taking part in this study?

It is important that you answer all the questions asked by the therapist honestly and completely. If your condition or circumstances change during the study, you must tell the therapist. You must inform your therapist immediately if you make any changes to any of your current treatments. It is important that you follow your therapist's instruction throughout the entire of the study.

7. What kind of treatment will I receive after my participation in the trial?

After the trial, you will continue receive necessary treatment as usual. Whether you complete the study or withdraw early, your therapist will discuss the best alternatives for your future treatment with you.

8. What are the potential risks and side effects of being in this study?

The potential risks of participating in this study is:

a) Wear of the splint may cause pressure injury to the skin

The researcher of this study will take necessary precaution and careful supervision throughout the entire process of splint fabrication to minimise these potential risks. You are advised to communicate and inform the researcher immediately if you have any concern of the above risks.

9. What if I am injured during this study?

If you are injured as a result of being in this study, you should contact the therapist researcher. If necessary, you will receive treatment by a doctor.

10. What are the benefits of being in this study?

Similar intervention will be given to participants and non-participants. There will be no direct benefit to you for your participation in this study. However, we hope that the information obtained from this study could help us improve the management of trigger finger in future.

11. Do you have to take part?
Your participation in this study is voluntary. You do not have to be in this study if you do not want to. By signing the informed consent form, this would mean that you have agreed to participate in this research. You may also refuse to answer any questions you do not want to answer. If you volunteer to be in this study, you may withdraw from it at any time. If you withdraw, any data collected from you up to your withdrawal will still be used for the study. Your refusal to participate or withdrawal will not affect any medical or health benefits to which you are otherwise entitled.

12. Who is funding the research?
This study is funded by the researcher herself. No external funding or grant is received. Therefore, no payment will be given for you for participating in this study. You do have to pay for the visit to clinic. All the splints and procedures you received are under the government hospital services. It is not charged for Malaysia citizens based on Medical Regulation 2017.

13. Do you cover by insurance while participating this study?
There is no insurance coverage for patients who participated in this study. Insurance for participants is not required because splinting is a regular procedure and standard intervention that often provided by occupational therapists to patients with trigger finger.

14. Can this study or my participation be terminated early?
The researcher may stop the study or your participation at any time due to the concerns of your safety. The study also may terminate when there is inadequate patients' enrolment, date and record are inaccurate and/or incomplete, or not adherence to the protocol while carrying out the study. If the study is stopped early for any reason, you will be informed, and arrangements will be made for your future care.

15. Will my medical information be kept private?
All your information obtained in this study will be kept and handled in a confidential manner, in accordance with applicable laws and/or regulations. When publishing or presenting the study results, your identity will not be revealed without your expressed consent. Individuals involved in this study and in your medical care, qualified auditors, and governmental or regulatory authorities may inspect and copy your medical records, where appropriate and necessary. You will not be allowed to view your personal information and study data, as the data will be consolidated into a database. After completion of the research, you will be informed of the study findings.

The data from this study will be made into a report which may be published. Once the data have been gathered and the research completed, the raw materials will be kept in the locked file cabinet in the office of the researcher. Electronic data will be securely kept using password. Access to the data is only by the researcher. After 5 years, the material will be shredded and discarded. The data will be reported in a collective manner with no reference to an individual.

16. Who should I call if I have questions?

If you have any questions about the study or if you think you have a study related condition and you want information about treatment, please contact the following researchers:

Dr. Chai Siaw Chui
Main Supervisor
Occupational Therapy Programme
Faculty of Health Sciences
Universiti Kebangsaan Malaysia

Ms. Leong Li Xian
Master's Candidate
Occupational Therapy Programme
Faculty of Health Sciences
Universiti Kebangsaan Malaysia

If you have any questions about your rights as a participant in this study, please contact: The Secretary, Medical Research & Ethics Committee, Ministry of Health Malaysia, at telephone number 03-xxxxxxx.

PATIENT INFORMED CONSENT FORM (ENGLISH)

Title of Study: Randomized Control Trial: Relative Motion Splint Versus Metacarpophalangeal Joint Blocking Splint in the Management of Trigger Finger, Which Bring Better Outcome and Better Wearing Experience?

By signing below, I confirm the following:

- I have been given oral and written information for the above study and have read and understood the information given.
- I have had sufficient time to consider participation in the study and have had the opportunity to ask questions and all my questions have been answered satisfactorily.
- I understand that my participation is voluntary, and I can at any time free withdraw from the study without giving a reason and this will in no way affect my future treatment. I am not taking part in any other research study currently. I understand the risks and benefits, and I freely give my informed consent to participate under the conditions stated. I understand that I must follow the researcher's instructions related to my participation in the study.
- I understand that study staff, qualified auditors, and governmental or regulatory authorities, have direct access to my medical record in order to make sure that the study is conducted correctly and the data are recorded correctly. All personal details will be treated as STRICTLY CONFIDENTIAL.
- I will receive a copy of this subject information/informed consent form signed and dated to bring home.

Subject:

Signature: I/C number:

Name: Date:

Investigator conducting informed consent:

Signature: I/C number:

Name: Date:

Impartial witness:

Signature: I/C number:

Name: Date:

DATA COLLECTION FORM
DEMOGRAPHIC QUESTIONAIIRE

ID		
Age		
Gender	F / M	
Diagnosis		
Associated medical conditions		
Hand dominance	R / L	
Finger involved	Right hand	Left hand
	Index finger ( )	Index finger ( )
	Middle finger ( )	Middle finger ( )
	Ring finger ( )	Ring finger ( )
	Little finger ( )	Little finger ( )
Duration of signs and symptoms		

STAGES OF STENOSING TENOSYNOVITIS (SST)

Stage	Finger movement	Finger involved	
		Right	Left
Stage 1	Normal		
Stage 2	Uneven		
Stage 3	Triggering or clicking or catching		
Stage 4	Locking of finger in flexion or extension, which can be unlocked by active finger movement		
Stage 5	Locking of finger in flexion or extension, which requires application of passive movement to unlock		
Stage 6	Locked finger in flexion or extension		

NUMBER OF TRIGGERING EVENTS IN TEN ACTIVE FISTS

Finger involved	Number of triggering events
Single finger	
Multiple fingers<br><br>(worst finger's involment)	

VISUAL ANALOG SCALE (WEEK-1 / WEEK-6)

PAIN

How much pain do you have when you are at rest?
(Berapa banyak kesakitan yang adas emasa anda sedang rehat?)

No pain
(Tiada kesakitan)

Worst pain
(Kesakitan yang paling teruk)

How much pain do you have when you are performing activities?
(Berapa banyak kesakitan yang ada semasa anda melakukan aktiviti?)

No pain
(Tiada kesakitan)

Worst pain
(Kesakitan yang paling teruk)

Notes (Catatan): ………………………………………………………………………………

VISUAL ANALOG SCALE (WEEK-1 / WEEK-3 / WEEK-6)

SPLINT WEARABILITY: COMFORT

Are you comfortable with splint that you are wearing at rest?
(Adaka handa selesa dengan splint yang anda pakai semasa anda sedang berehat?)

Not at all comfortable (Tidak selesa langsung)	Extremely comfortable (Sangat selesa)

Are you comfortable with splint that you are wearing while performing activities?
(Adakah anda selesa dengan splint yang anda pakai semasa anda melakukan aktiviti?)

Not at all comfortable (Tidak selesa langsung)	Extremely comfortable (Sangat selesa)

Notes (Catatan): ……………………………………………………………………

SPLINT WEARABILITY: SATISFACTION

Are you satisfied with splint that you are wearing at rest?
(Adakah anda berpuas hati dengan splint yang anda pakai semasa anda sedang rehat?)

Not at all satisfied (Tidak berpuas hati langsung)	Extremely satisfied (Sangat berpuas hati)

Are you satisfied with splint that you are wearing while performing activities?
(Adakah anda berpuas hati dengan splint yang anda pakai semasa anda melakukan aktiviti?)

Not at all satisfied (Tidak berpuas hati langsung)	Extremely satisfied (Sangat berpuas hati)

Notes (Catatan): ……………………………………………………………………
